# Interspecies interactions drive bacterial proteome reorganization and emergent metabolism

**DOI:** 10.1038/s41559-026-03030-4

**Published:** 2026-03-31

**Authors:** Stephan Kamrad, Simran K. Aulakh, Simone Mozzachiodi, Sonja Blasche, David Scheidweiler, Arianna Basile, Rui Guan, Rob Bradley, Naomi Iris van den Berg, Michael Mülleder, Markus Ralser, Kiran R. Patil

**Affiliations:** 1 The Medical Research Council Toxicology Unit, University of Cambridge, Cambridge, UK; 2 Core Facility High-Throughput Mass Spectrometry, Charité Universitätsmedizin, Berlin, Germany; 3 Department of Biochemistry, Charité Universitätsmedizin, Berlin, Germany; 4 Centre for Human Genetics, Nuffield Department of Medicine, University of Oxford, Oxford, UK; 5 Max Planck Institute for Molecular Genetics, Berlin, Germany; 6 Department of Biochemistry, University of Cambridge, Cambridge, UK

**Keywords:** Microbial communities, Microbial ecology, Emergence

## Abstract

Species in microbial communities need to stave off competition and capitalize on new resources that become available because of metabolic activities of others. However, intra-cellular molecular changes that underpin these responses are understudied, preventing mechanistic insights into community function and dynamics. Here we analyse proteomic and metabolomic responses in 104 pairwise co-cultures of 15 gut bacteria, spanning a diversity of ecological interactions from competition to mutualism. We find that molecular responses to co-culturing are substantial, with typically 50% of the quantified proteome changing in at least one co-culture, jointly influenced by genome size, species abundance and pH. Even closely related species and orthologue proteins show different expression profiles in response to the same partner, indicating functional diversification at both protein and species level. Small-molecule transport and carbon metabolism are among the most responsive processes, indicating pervasive metabolic interactions. Using metabolomics, we identify likely cross-fed metabolites, emergent polyamine metabolism and niche partitioning in amino acid utilization. Overall, our study uncovers how bacteria respond to the presence of other species through extensive remodelling of their proteome and metabolome.

## Abstract (web summary)

An analysis of the responses of 15 diverse human gut bacteria in more than 100 pairwise co-cultures reveals extensive remodelling of the bacterial proteome and metabolome in response to the presence of other species.

## Main

Comprehensive compositional catalogues of diverse microbial communities important for health and the environment are increasingly becoming available^1–6^. An outstanding challenge is to understand the mechanisms underlying interspecies interactions that determine the compositional and functional dynamics of these communities in response to biotic and abiotic perturbations^7,8^. However, molecular analysis at the community scale is challenging owing to the compositional and dynamical complexity of natural communities. Reductionist (‘bottom-up’) approaches and in particular pairwise interaction analyses^9–16^ are therefore instrumental in unravelling mechanisms shaping community-level emergent functions^17,18^.

Herein, we use gut bacterial isolates to probe the molecular basis of interspecies interactions. Ecological interactions among gut bacteria comprise complex competitive and co-operative interactions^19–23^. For example, many species collectively contribute to the biosynthesis of short chain fatty acids (SCFAs)^24,25^ and tryptophan-derived metabolites^26^. Gut bacteria therefore present an attractive model to study how community outputs emerge from the interactions of microbial species and their metabolic activities.

A key question is how enzyme abundances and metabolite production are regulated within the community context. Only a few studies^27–30^ have systematically investigated functional interactions at the level of gene expression, revealing which subset of the genetic (for example, enzymatic) repertoire is realized in specific conditions^31^. In this context, proteomic measurements are more accurate readouts of functional state than transcriptomics as they capture post-transcriptional regulation and provide direct measurements of the molecules that carry out the function, for example, a metabolic reaction. Recent advances in proteomics precision, depth and throughput^32,33^ enable the application of proteomics to species mixtures^34–40^. Previous small-scale studies investigating the regulation of gene expression in synthetic gut bacterial communities in vitro^41^ or in mice^42,43^ revealed substantial proteome remodelling and niche specialization depending on community context. This is further supported by a microarray-based meta-transcriptomics study of human stool samples^44^. Overall, while the importance of species interactions in shaping microbial gene expression and metabolism are widely acknowledged, there is a striking lack of large-scale studies, limiting the current understanding of molecular processes underpinning interspecies interactions.

Herein, we employ systematic proteomics and metabolomics of binary co-cultures to map physiological changes that emerge between 104 pairs of 15 diverse human gut bacteria. A substantial fraction of proteins responds to the presence of at least one other species, in particular transporters, metabolic enzymes as well as many unannotated/understudied proteins. We complement these data with metabolomics to identify putative exchanged and emergent metabolites. Our systematic approach constitutes a framework and resource to uncover the mechanistic basis of interspecies interactions.

## Results

### Combinatorial co-cultures for the systematic molecular characterization of species interactions

**Fig. 1:**
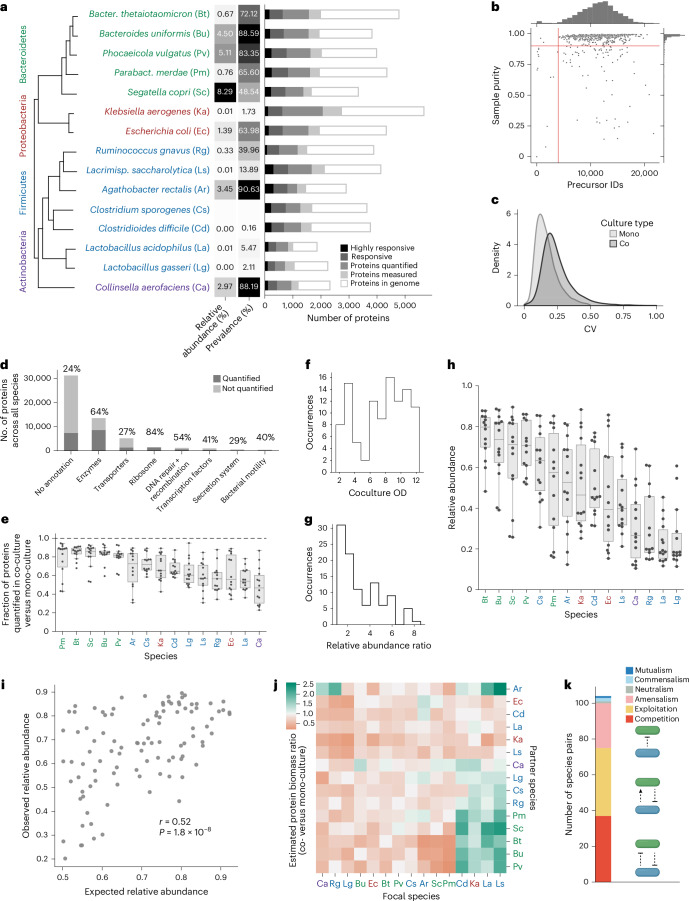
Extensive regulation of proteomes in pairwise co-cultures of human gut bacteria. **a**, In total, 15 bacterial strains were selected on the basis of abundance, prevalence and genetic diversity. Proteomes of all pairwise co-cultures were obtained by DIA-proteomics, The phylogenetic tree was generated with OrthoFinder using protein sequences. Bacterial phyla are highlighted in colours. A two-letter code for each species used throughout this manuscript is indicated in parentheses. The mean relative abundances and prevalences are shown in the heat map and were obtained from a curated set of metagenomic studies^6^. The bar chart illustrates the number of proteins encoded in each genome, the proteins measured in our dataset (supported by ≥1 high-quality peptide in ≥1 biological replicates), the quantified proteins (≥2 peptides in ≥2 biological replicates), the responsive proteins (quantified proteins that are differentially abundant in at least one co-culture; *P*_adj_ < 0.05, abs(log_2_(fold change)) >0.5, limma FDR-adjusted moderated two-sided *t*-test) and highly responsive proteins (differentially abundant in at least four conditions). **b**, The quality control of proteomics samples. In total, 768 samples were acquired using dia-PASEF and searched against a combined predicted spectral library of all species. The median number of identified precursors was 17,625. Sample purity was estimated as the fraction of identified precursors assigned to the species expected in the sample. Overall, 608 samples with >4,000 identified precursors and a purity of >90% were taken forward (upper-right quandrant of intersecting red lines). **c**, Dataset-wide distributions of CV values across mono-cultures (median of 16%, reflecting biological/technical noise) versus co-culture conditions (median of 27%, indicating substantial additional variation in response to co-culture partners). **d**, The coverage of different functional protein classes (BRITE) by our dataset that includes 31,127 proteins across all species. **e**, The fraction of proteins quantified in co-culture versus mono-culture controls. Each datapoint represents a co-culture condition (*n* ≈ 4 biological replicates). Good data completeness is maintained across co-cultures despite increasing sample complexity. Box plot elements are defined as: centre line, median; box limits, upper and lower quartiles; whiskers, 1.5× interquartile range; and points, outliers. **f**, The distribution of total co-culture biomass in OD_600_ units at the time of cell collection (24 h). **g**, The distribution of relative abundance ratios (major species/minor species) across co-cultures. Relative abundance was estimated from peptide intensities (Methods). **h**, The relative abundance of each species across 14 co-culture conditions. Each point represents the mean of *n* ≈ 4 biological replicates. Some species were consistently more abundant than others, although no single species was always or never the major partner. Box plot elements are defined as: centre line, median; box limits, upper and lower quartiles; whiskers, 1.5× interquartile range; and points, outliers. **i**, The relative abundance in co-cultures predicted using the ratio of mono-culture OD_600_ correlates poorly with observed relative abundance across all species pairs, that is, species with similar mono-culture OD_600_ values can form co-cultures with widely varying ratios, indicating the prevalence of interactions. *r*, Pearson correlation coefficient, *P*, two-sided *P* value of the null hypothesis *r* = 0. **j**, Interspecies growth interactions (absolute protein biomass in co-/mono-culture, log_2_-transformed) of partner species (*y* axis) on focal species (*x* axis). Negative interactions dominate, but a small group of species (*L. saccharolytica*, *L. acidophilus*, *C. difficile* and *K. aerogenes*) benefits from the presence of certain Bacteroidetes and *A. rectalis*. Grey indicates that data are not available. **k**, The types of ecological interactions found across 104 species pairs. A mean change in growth of >10% was considered a positive/negative effect. Competition, exploitation and amensalism are the predominant forms of ecological interactions across the dataset. Bt, *Bacteroides thetaiotaomicron*; Bu, *Bacteroides uniformis*; Pv, *Phocaeicola vulgatus*; Pm, *Parabacteroides merdae*; Sc, *Segatella copri*; Ka, *Klebsiella aerogenes*; Ec, *Escherichia coli*; Rg, *Ruminococcus gnavus*; Ls, *Lacrimispora saccharolytica*; Ar, *Agathobacter rectalis*; Cs, *Clostridium sporogenes*; Cd, *Clostridioides difficile*; La, *Lactobacillus acidophilus*; Lg, *Lactobacillus gasseri*; Ca, *Collinsella aerofaciens*. Icons created in BioRender; Kamrad, S. https://biorender.com/13dsg6p (2026). Source data

To study the functional adaptation of bacterial gene expression and metabolism in response to other community members, we selected 15 human gut bacterial strains (Fig. 1a and Supplementary Table 1). These include abundant and prevalent commensal species from four phyla (Firmicutes, Bacteroidetes, Proteobacteria and Actinobacteria), as well as two common pathogens, *Clostridioides difficile* and *Klebsiella aerogenes*, and two probiotics, *Lactobacillus acidophilus* and *Lactobacillus gasseri*. This diverse set includes species with complementary nutrient requirements and known cross-feeding interactions^19^, as well as understudied interactions such as those between probiotics and resident microbiota. Strains were cultured in a pairwise combinatorial design in 96-well plates, resulting in 608 samples (after quality control) across 104 species pairs (*n* ≈ 4 biological replicates, plus *n* ≈ 16 biological replicates of each mono-culture as controls). At the stationary growth phase, optical densities (ODs) at 595 nm were determined and cell pellets and supernatants were harvested and processed for proteomic and metabolomic measurements by liquid chromatography tandem mass spectrometry.

**Extended Data Fig. 1:**
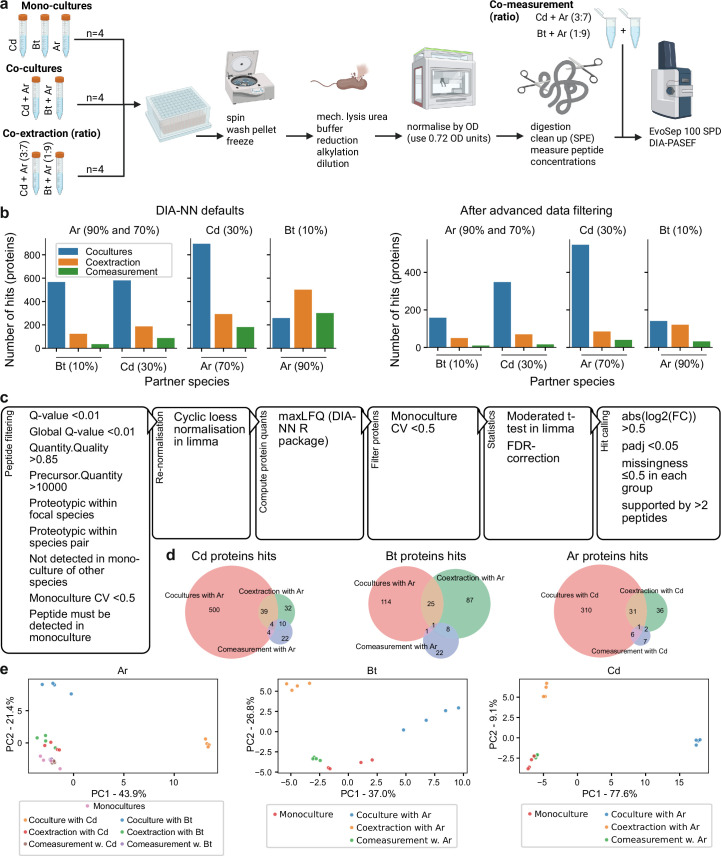
Benchmark dataset controls for matrix effects. (**a**) Overview of benchmark experiment workflow. Three co-cultures containing gram-positive and -negative species at different ratios spanning the previously observed range (1:1 to 1:9) were measured in quadruplicates, as well as the same number of corresponding mono-cultures. To isolate LC–MS matrix effects in the absence of biological proteome changes, we mixed mono-culture samples in the same ratio (co-measurement controls). Additionally, we mixed mono-cultures in the same ratio before protein extraction (co-extraction controls) to detect potential artefacts introduced during sample preparation. Created in BioRender. Kamrad, S. (2026) https://BioRender.com/13dsg6p. (**b**) Number of hits with DIA-NN default parameters (that is, using protein quantities provided by DIA-NN without additional filtering, right) versus after filtering (see panel C, right). Hits are defined relative to mono-culture controls, abs(log_2_(co-/mono-culture)>0.5 and p_adj_ < 0.05. (**c**) Implemented data filtering steps which successfully remove most coextraction and co-measurement artefacts. (**d**) Overlap of hit proteins across co-culture and control conditions. (**e**) Principal component analysis of proteome profiles, each datapoint represents an individual sample. Schematic in **a** created in BioRender; Kamrad, S. https://biorender.com/13dsg6p (2026).

**Extended Data Fig. 2:**
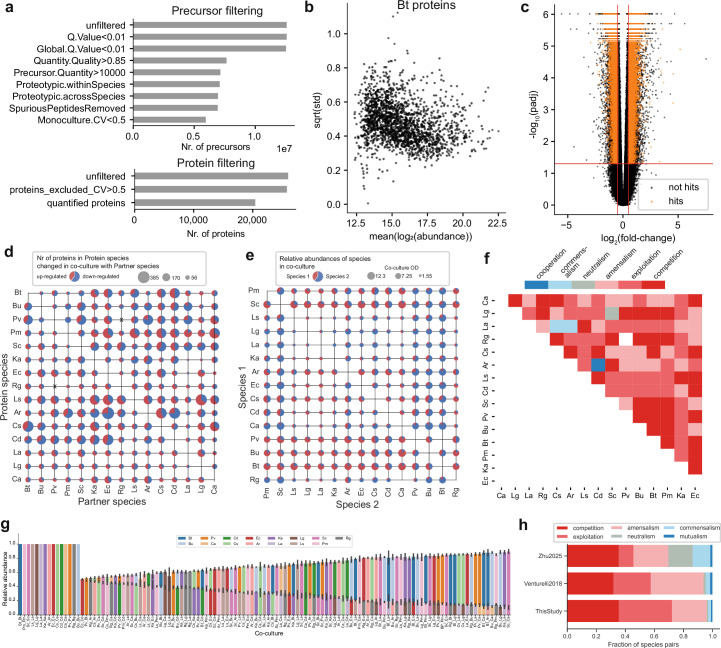
Proteomics data processing and ecological interactions. (**a**) Illustration of precursor- and protein-level filtering. Bar widths reflect the number of remaining precursors/proteins after each filtering step. This plot aggregates data from the 15 individual DIA-NN runs (one per species). Each run was processed separately. Precursors were filtered as shown from top to bottom (also see Methods). The filtered precursors were then re-normalised across samples and protein quantities were computed with maxLFQ. Proteins were filtered based on mono-culture CV only. (**b**) Differential abundance was assessed using empirical Bayes statistics as implemented in *limma*. This method squeezes the variance towards a global abundance versus variance trend, which is illustrated here exemplarily for the species *B. thetaiotaomicron*. (**c**) A fold-change cut-off of abs(log_2_(co-/mono-culture))>0.5 and a significance cut-off of p_adj_ < 0.05 (species-wise FDR-corrected, two-sided moderated t-test) was applied to the differential abundance statistic as illustrated in this volcano plot which aggregates data for all 15 species. 14.07% of the tested interactions were significant. (**d**) Number of hits in each co-culture. The size of each pie chart reflects the overall number of hits, the distribution of up- versus down-regulated proteins is shown in colours. (**e**) Relative abundance of species across co-culture conditions. The size of the pie chart reflects the total co-culture OD. (**f**) Ecological interaction types for all co-cultures. (**g**) Relative abundances for all 104 co-culture pairs, bar heights indicate the mean, errorbars indicate the standard deviation (n ≈ 4 biological replicates per co-culture). (**h**) Comparison of frequencies of ecological interaction types with other studies. Venturelli et al. 2018^11^ used liquid cultures in Anaerobic Basal Broth, while Zhu et al. 2025^16^ used solid mGAM medium. Source data

To determine cellular proteomes, we used a throughput-optimized workflow on an Evosep One chromatography system with data-independent acquisition (DIA) by parallel accumulation serial fragmentation (dia-PASEF^45^) on a trapped ion mobility spectrometry–time of flight (timsTOF) mass spectrometer. Data were analysed with DIA-NN^46^, using a library-free search with a combined fasta-database (Supplementary Tables 2 and 3). The workflow was benchmarked using a controlled experiment to check for matrix effects and possible artefacts introduced by co-extracting bacterial species (Methods; Extended Data Fig. 1). Out of 768 samples for which data was acquired, 106 samples were excluded because they had fewer than 4,000 precursor identifications or more than 10% of identified precursors were originating from species other than those expected in the sample (Fig. 1b). In mono-cultures, after quality filtering (Methods; Extended Data Fig. 2a), we quantified over 1,300 proteins (37% of the proteins encoded in the genome) per species on average (Fig. 1a). The median, protein-level coefficient of variation (CV) was 15% across mono-culture controls and 22% across co-cultures, indicating low experimental noise levels and substantial biological responses in co-culture (Fig. 1c). The number of identified proteins compares favourably with other current, state-of-the art studies in bacterial model species^47,48^ and indicates high proteome coverage despite short analysis times. Our dataset, such as other proteomic datasets, is expected to capture most of the proteome by mass, since highly abundant proteins are much more likely to be measured. For example, Schmidt et al.^49^ estimated that the 55% of *Escherichia coli* open reading frames detected in their dataset cover 95% of the proteome by mass.

At the functional level, we obtained >60% coverage of annotated enzymes and ribosomal proteins, >40% coverage of annotated transcription factor and motility proteins but comparatively sparser (<30%) coverage of membrane proteins (transporters and secretion systems) and unannotated proteins (Fig. 1d). In co-cultures, we typically detected a much higher number of proteins overall, but the coverage per individual species declined slightly to 55–84% of the mono-culture proteins (Fig. 1e, first and third quartile). This is expected as the abundance of peptides from each species is lower and the sample complexity is higher for two-species meta-proteomes in comparison with single-species proteomes. In summary, we collected a systematic, broad and deep proteomics dataset of bacterial co-cultures.

### Diverse ecological interactions emerge in co-cultures

First, we leveraged the proteomics data and co-culture ODs to determine abundances of individual species in co-culture. OD values of co-cultures, representing total biomass, typically ranged from 3.1 to 9.7 (first and third quartile, respectively) with no clear distribution pattern (Fig. 1f). For all co-cultures, we computed the absolute abundances of each member species on the basis of species-specific peptide intensities and the OD of the co-culture (Methods; ref. ^29^). In the majority of co-cultures (64%), one species was substantially more abundant than the other (>2-fold), although in only a small minority (19%) the ratio was more than 5-fold (Fig. 1g). Although no single species was always or never the dominant partner across all co-cultures, a few species were usually the major species (the Bacteroidetes *Phocaeicola vulgatus*, *Bacteroides uniformis*, *Bacteroides thetaiotaomicron* and *Segatella copri*), and a few others were usually the minor species (the *Lactobacilli L. gasseri* and *L. acidophilus*, as well as *Collinsella aerofaciens* and *Ruminococcus gnavus*) (Fig. 1h). The growth of a species in mono-culture (final OD) was able to explain some of the relative abundance in co-culture (*r* = 0.52), but for species with similar ODs in mono-culture the prediction was poor (Fig. 1i), indicating prevalent interspecies interactions.

**Extended Data Fig. 3:**
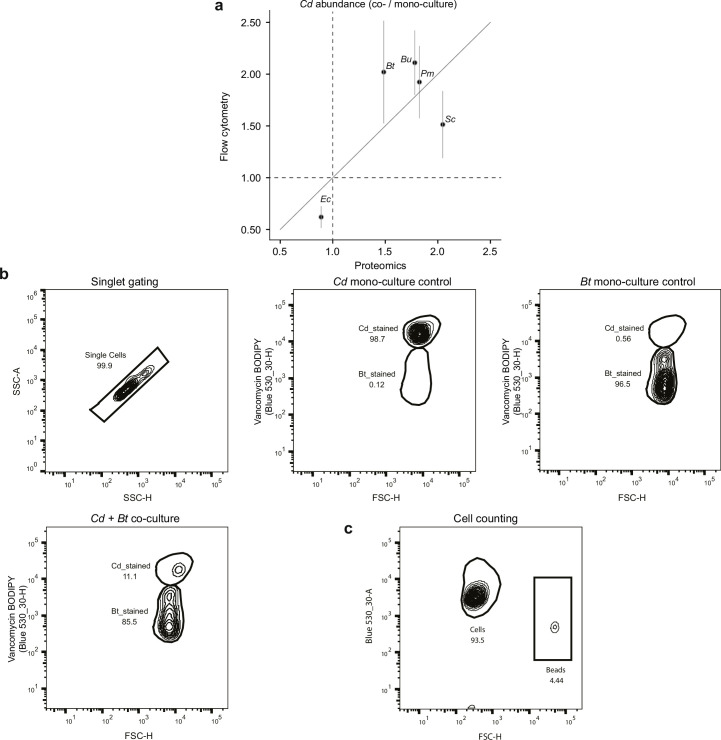
Quantitative flow cytometry. (**a**) Ratio of abundance of *C. difficile* in co- versus mono-culture with 5 selected partner species obtained via main proteomics experiment (x-axis, subset of data shown in Fig. 1j) and quantitative flow cytometry (y-axis). Flow cytometry was performed on n = 3 biological replicates, errorbars indicate the standard deviation. This independent measurement confirms that the absolute abundance of *C. difficile* increases in co-culture with the selected Bacteroidetes but decreases in co-culture with *E. coli*, as indicated in the main proteomics experiment. (**b**) Representative plots illustrating the gating strategy used for relative quantification of species via stain which distinguishes gram-positive/negative cells (vancomycin-biodipy conjugate). (**c**) Representative plot illustrating the gating strategy used for absolute quantification of bacterial cells via bead spike in.

The majority (137/223) of growth interactions across all co-cultures were negative (>10% decreased absolute mean abundance in co-culture versus mono-culture) consistent with the resource limitation in batch cultures, yet a substantial minority (42/223) was positive (>10% increase in growth) and the rest (44/223) neutral (Fig. 1j, Extended Data Fig. 2e–g and Supplementary Table 4). We identified four phylogenetically diverse species, *Lacrimispora saccharolytica, C. difficile, L. acidophilus* and *K. aerogenes*, which benefit from the presence of Bacteroidetes and *Agathobacter rectalis*. The magnitude of these interactions is substantial; for example, *L. saccharolytica*, which had a biomass of 1.38 ± 0.06 OD units in mono-culture, grew to 3.57 ± 0.2 in the presence of *A. rectalis* and to 3.18 ± 0.21 in the presence of *S. copri*. We independently validated the growth promotion of *C. difficile* by four Bacteroidetes species using quantitative flow cytometry (Extended Data Fig. 3).

Across all the co-cultures, 35% of pairs could be classified as competitive (absolute abundance of both species lower than in their respective mono-cultures) and 24% as amensal (one species inhibited, the other unchanged) (Fig. 1k and Extended Data Fig. 2e). However, the most common (36%) interaction type was exploitation (where one species increased in abundance, the other decreased). Together with two commensal and one mutualistic species pairing, 39% of pairings involve a positive outcome for a member species. These results are in good agreement with other methodologically complementary studies (different readouts and media) of pairwise interactions among gut bacteria^11,16^ (Extended Data Fig. 2h) Overall, our co-culture model spanned a broad diversity of growth behaviours and ecological interactions.

### Extensive proteome remodelling in the co-culture

To investigate the molecular changes associated with the various observed ecological interactions, we identified proteins that were differentially abundant in co-cultures, in comparison with the corresponding mono-cultures (log_2_-transformed absolute fold change >0.5, false discovery rate (FDR)-adjusted *P* value (*P*_adj_) <0.05, moderated *t*-test via limma, ≥2 measured (designated as ‘non-NA’) replicates and ≥2 unique matching precursors; Extended Data Fig. 2b–d and Supplementary Table 2).

Between 30% (*K. aerogenes*) and 70% (*A. rectalis*) of quantified proteins (median, 49%) were differentially expressed in at least one co-culture (Fig. 1a). Overall, the proteome response was highly specific to the co-culture partner (38% of responsive proteins only differentially abundant in a single co-culture); however, we also identified common responsive proteins (hits in four or more conditions), which made up between 5.7% (*K. aerogenes*) and 27% (*A. rectalis*) of quantified proteins (median, 14%).

### Physiological factors underlying strength of proteome response in co-cultures

In the search of a mechanistic basis and general principles underlying the proteome changes observed in co-culture, we investigated a range of factors ranging from intrinsic properties of a species, such as its proteome size, and extrinsic factors, such as pH change (Supplementary Table 5).

**Fig. 2:**
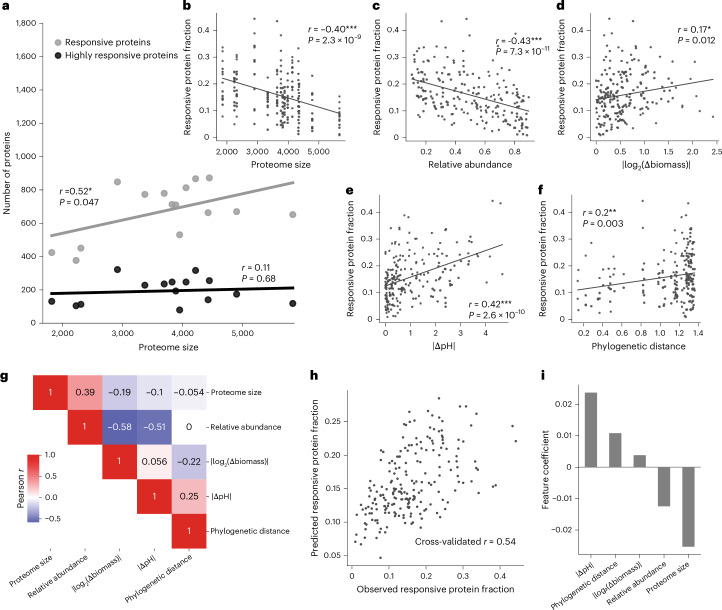
Broad factors underlying the magnitude of proteome response. **a**, The number of responsive and highly responsive proteins (hit in at least four co-culture conditions) dependent on proteome size at the species level. The number of responsive proteins increases with genome size, although not at the same rate. The number of highly responsive proteins is not correlated with genome size. **b**, The responsive proteome fraction (number of hits/number of proteins in proteome, *y* axis) across all 208 datapoints (104 co-cultures, from the perspective of each species) versus proteome size (number of proteins encoded in the genome). **c**, As in **b** but for relative abundance (fraction of protein biomass in co-culture attributable to species). **d**, As in **b** but for the absolute log_2_-transformed fold change of the biomass reached in co-culture versus mono-culture (|log_2_(Δbiomass)|), reflecting ecological interactions with the partner species. **e**, As in **b** but for the absolute change in pH between the co-culture and the mono-culture (|ΔpH|). **f**, As in **b** but for the phylogenetic distance between the co-culture partners, obtained from the tree shown in Fig. 1a. **g**, A heat map indicating Pearson correlations between the factors shown in **c**–**f**. **h**, A multiple linear regression model based on the factors from **c**–**f** was used to predict the responsive proteome fraction. Leave-one-out cross-validation was used to assess the ability of the model to predict unseen interactions. **i**, Model coefficients indicate the importance of each shown feature in predicting the magnitude of the proteome response. The proteome size of the focal species and changes in pH were identified as major drivers, followed by relative abundance. Conversely, changes in growth and phylogenetic distance between the partner species had a minor impact. For **a**–**f** and **h**, the Pearson correlation coefficient is indicated in the plot and fitted lines were obtained by ordinary least-squares regression. Significance is denoted by ***P < 0.001, **P < 0.01, *P < 0.05 (two-sided test if *r* = 0). Source data

**Extended Data Fig. 4:**
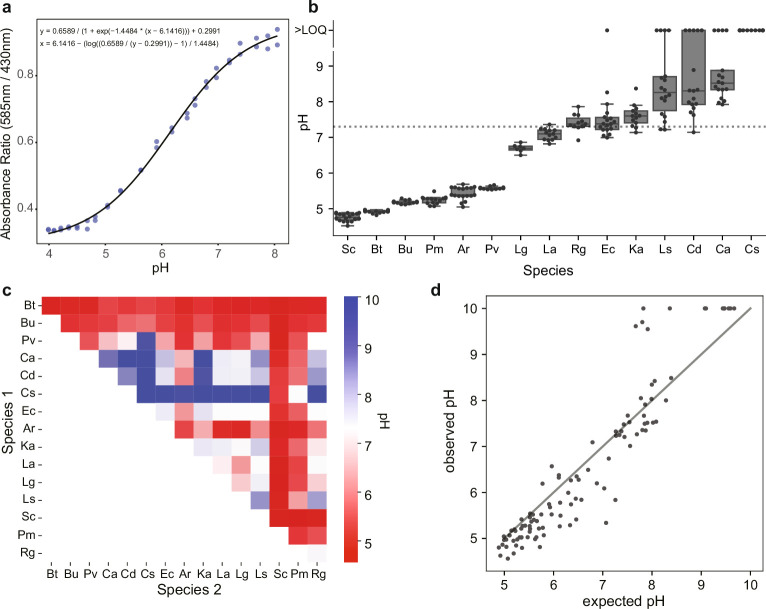
pH of co-cultures. (**a**) Calibration curve of high-throughput pH assay using litmus dye. The line shows the fitted sigmoidal calibration curve (formula shown in the plot). (**b**) Mono-culture pH values. LOQ = limit of quantification. The dotted line indicates the pH of fresh media. Following convention, box-plot elements are defined as follows: centre line, median; box limits, upper and lower quartiles; whiskers, 1.5× interquartile range. (**c**) Mean co-culture pH values (n ≈ 4 biological replicates per co-culture). (**d**) Observed versus expected (average of mono-culture pH, weighted by relative abundance of species in co-culture). The line represents the expected x = y relationship.

(1) Proteome size: the number of proteins encoded in the genomes of the selected species varied more than twofold—between 5,670 for *K. aerogenes* and 1,859 for *L. acidophilus* (median 3,820) (Fig. 1a). Proteome size was correlated with the total number of responsive proteins across co-cultures but not the number of highly responsive proteins (Fig. 2a). Thus, across diverse species, there is a consistently large set of approximately 200 proteins that is highly regulated in response to other species. As proteome size increases, the number of regulated proteins increases at a lower rate, resulting in a negative correlation between proteome size and the responsive protein fraction (the number of hits divided by the number of quantified proteins) (Fig. 2b). (2) Relative abundance: **w**e note an appreciable correlation between relative abundance and the fraction of differentially expressed proteins (Fig. 2c), potentially because relatively low abundant species are more exposed to the other species’ cells, secreted proteins and metabolites than their own. (3) Growth: changes in growth are known to be linked with proteome changes^49,50^. Indeed, the responsive protein fraction correlates with the magnitude of change in the biomass of the species in co-culture compared with mono-culture (Fig. 2d). However, even low abundant species can have a pronounced effect on the protein expression of high abundant species. For example, the dataset-wide strongest response was observed in *A. rectalis* when co-cultured with *E. coli* (385 responding proteins), although *A. rectalis* was the strongly dominant species (relative abundance of 80.7%). (4) pH: bacterial metabolism commonly produces overflow metabolites such as SCFAs and other organic acids that alter the pH of the surrounding environment. We assessed the pH of the co-cultures at the time of proteomic and metabolomic sampling using an indicator dye (Methods). In mono-cultures, we observed strong media acidification by Bacteroidetes and *A. rectalis* (Extended Data Fig. 4), compatible with their known capacity for producing organic acids during carbohydrate fermentation^51,52^. The Clostridia *C. sporogenes* and *C. difficile* increased the pH, attributable to ammonia production during Stickland metabolism of amino acids^53,54^. *L. saccharolytica* and *C. aerofaciens* likewise raised the pH. In co-cultures, strong changes in pH compared with the mono-culture were associated with an increased number of differentially expressed proteins (Fig. 2e). (5) Phylogenetics: finally, phylogenetic distance between co-culture partners was weakly correlated with the responsive proteome fraction (Fig. 2f).

To gauge the combined influence that these factors hold, we constructed a multiple linear regression model and performed leave-one-out cross-validation (Fig. 2g). This model achieved a Pearson *r* of 0.54 in predicting the responsive protein fraction (Fig. 2h). Analysis of the model coefficients revealed changes in pH and relative abundance as the most important drivers, followed by proteome size and phylogenetic distance (Fig. 2i). Thus, both intrinsic and extrinsic factors determine the proteomic response of a species to their co-culture partners.

### Carbohydrate metabolism and transport are highly responsive in co-culture

**Fig. 3:**
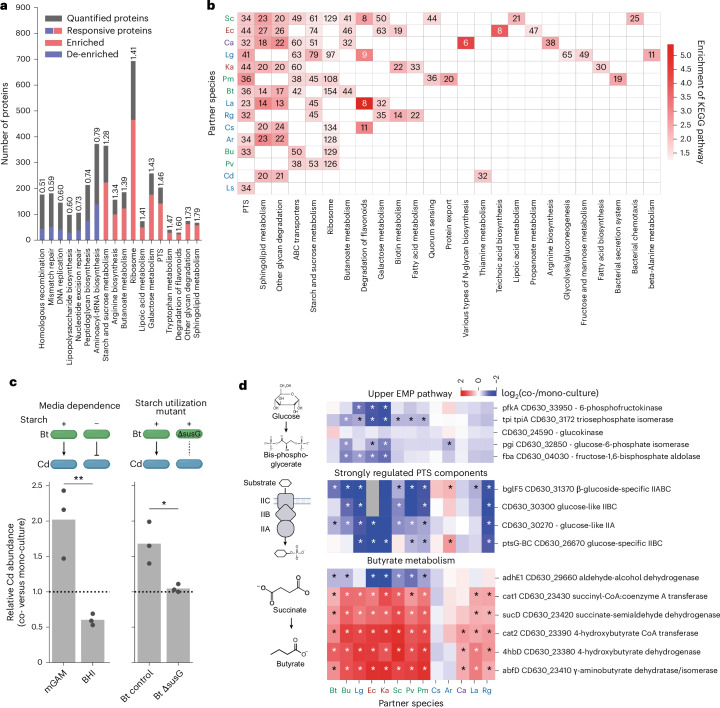
Functional analysis of proteome changes. **a**, A global enrichment analysis of KEGG pathways in responsive proteins (differentially expressed in at least one co-culture condition) versus all measured proteins as background. The numbers above the bars indicate the enrichment factor (term frequency in responsive proteins divided by background frequency). Only significantly enriched/de-enriched terms are shown (*P*_adj_ < 0.05, FDR-corrected two-sided Fisher’s exact test). **b**, The enrichment analysis of KEGG pathways in response to different partner species. Only significantly overrepresented terms are shown (*P*_adj_ < 0.05, FDR-corrected two-sided Fisher’s exact test). **c**, The stationary-phase abundance of *C. difficile* in co-culture with *B. thetaiotaomicron* divided by *C. difficile* abundance in mono-culture in the indicated conditions, captured by quantitative flow cytometry (Extended Data Fig. 3). In mGAM, the main medium used in this study, *B. thetaiotaomicron* promotes the growth of *C. difficile* (Fig. 1j), but in BHI medium the relative growth of *C. difficile* is significantly reduced (*P* = 0.0081, *n* = 3 biological replicates). A major difference between BHI and mGAM media is the absence of starch. This was further validated by the use of a *B. thetaiotaomicron* starch-utilization mutant (∆susG; Methods), which similarly reduces relative *C. difficile* abundance (*P* = 0.022, *n* = 3 biological replicates) in mGAM and appears unable to promote the growth of *C. difficile*. Significance is denoted by *P < 0.05, **P < 0.01, Student’s two-sided *t*-test. Bar heights represent means. **d**, The regulation of selected metabolic pathways in *C. difficile* across co-culture conditions. Top: upper Embden–Meyerhof–Parnas/glycolysis proteins. Middle: strongly regulated (sum(abs(log_2_(fold change))) >10) PTS components. The vast majority of these are annotated as glucose-specific (see Extended Data Fig. 5c for other PTS components). Bottom: enzymes of the pathway that converts succinate to butyrate. Source data

To identify biological processes differentially regulated in co-culture versus mono-culture, we used a sequence-based search to map proteins to Kyoto Encyclopedia of Genes and Genomes (KEGG) orthologous groups and retrieve associated functional annotation (Methods). Globally, the set of responsive proteins (hit in at least one species-condition pair) was significantly enriched in 31 KEGG pathways (Fig. 3a) (*P*_adj_ < 0.05, FDR-corrected Fisher’s exact test). These included specific classes of nutrient transport machinery, namely the phosphotransferase system (PTS) and ATP-binding cassette transporters (ABC). Three other pathways involved in carbohydrate metabolism (butyrate metabolism, starch and sucrose degradation and ‘other glycan degradation’) were also globally enriched, indicating a central role for carbohydrate uptake and metabolism in mediating interspecies proteomic interactions. The set of responsive proteins were depleted in cellular housekeeping functions such as aminoacyl tRNA synthesis, RNA polymerase, homologous recombination and nucleotide metabolism, indicating that these core functions are buffered against communal perturbations.

**Extended Data Fig. 5:**
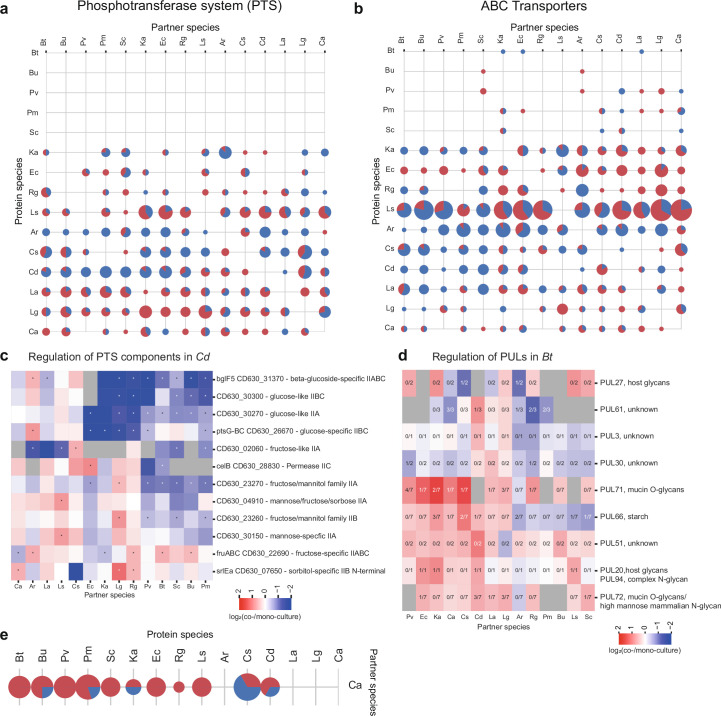
Differential protein abundance by functional groups. (**a**) Number of up- (red) and down-regulated (blue) proteins annotated to the phosphotransferase system (PTS) in each protein species in the presence of each partner species. The PTS system is absent in *Bacteroides*. (**b**) Number of up- (red) and down-regulated (blue) proteins annotated to the ATP-binding cassette transporters (ABC) in each protein species in the presence of each partner species. *L. saccharolytica* has an extraordinarily high number of (regulated) ABC transporters. (**c**) Regulation of PTS system components in *C. difficile*. Asterisks indicate significant interactions. (**d**) Regulation of polysaccharide utilising loci (PULs) in *B. thetaiotaomicron*. The colour scale reflects the abundance change of the most strongly changed protein annotated to that PUL. The fractions of proteins which are significantly up-/down-regulated are indicated in the heatmap. Only reliably measured (in at least 6 co-culture conditions) and strongly regulated (sum(abs(log_2_(fold-change))) > 4) PULs are shown. PUL annotation was obtained from (Ryan et al. 2024)^118^ which integrated data from refs. ^119–127^. (**e**) Number of proteins annotated to ‘Arginine biosynthesis’ which are up-/down-regulated in the presence of *C. aerofaciens*. Source data

Zooming in, we next asked which species trigger differential expression of which pathways in their co-culture partner (Fig. 3b). Many species induced changes in the PTS system (Extended Data Fig. 5a,c), ABC transporters (Extended Data Fig. 5b), starch and sucrose metabolism, sphingolipid metabolism and ‘other glycan degradation.’ *L. saccharolytica* had the highest number of (regulated) ABC transporters. Polysaccharide utilizing loci, however, were not strongly regulated in *B. thetaiotaomicron*, although data coverage of these membrane proteins was low, which may have masked some changes (Extended Data Fig. 5d). Other pathways were regulated in response to particular species. For example, arginine biosynthesis was often regulated in the presence of *C. aerofaciens* (Extended Data Fig. 5e), which depletes arginine from the media (see below). Overall, our data uncovered carbohydrate metabolism and nutrient transport pathways that are extensively regulated in response to the presence of other species.

**Extended Data Fig. 6:**
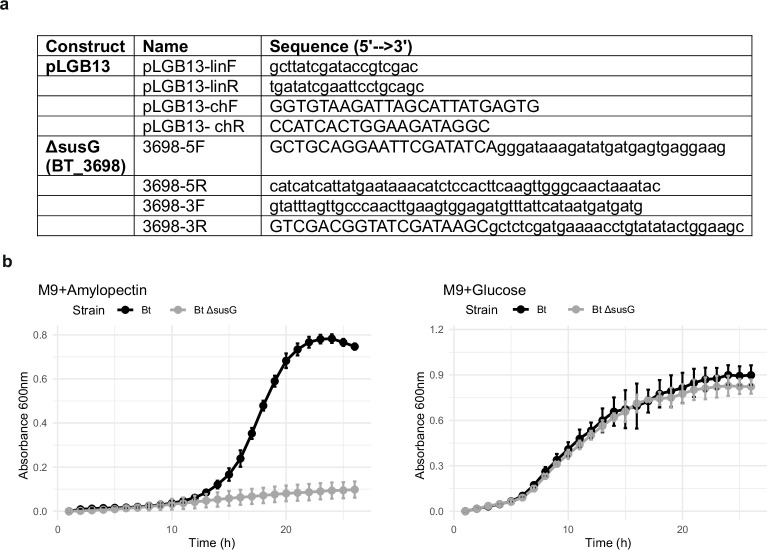
Starch utilization mutant construction and validation. (**a**) Table of primers used for constructing *B. thetaiotaomicron* susG mutant (Methods). (**b**) Growth curves in defined minimal medium containing amylopectin (left) and glucose (right) as the sole carbon source. Lines represent the mean, errorbars represent the standard deviation of n = 3 biological replicates.

As a major pathogen, ecological interactions of *C. difficile* with other gut bacteria have been studied intensely^14,55–57^. In one recent study^58^, *Bacteroides* and *Phocaeicola* were identified as suppressors of *C. difficile* when cultured continuously in Brain Heart Infusion (BHI) media with additional supplements, attributed partially to competition for amino acids. We were thus surprised to see a growth-promoting effect of several Bacteroidetes and *A. rectalis* on *C. difficile* (Fig. 1j). A major difference between the studies is the cultivation medium, and indeed we observed that *B. thetaiotaomicron* became growth-inhibiting instead of promoting in BHI medium (Fig. 3c). Since modified Gifu anaerobic broth (mGAM) contains substantial amounts (12 g l^−1^) of starch, we tested if starch metabolism by *B. thetaiotaomicron* affects *C. difficile* growth in co-culture by constructing a mutant lacking a key component of the starch utilization system (sus), the α-amylase susG (Methods; Extended Data Fig. 6). This mutant did not promote the growth of *C. difficile* (Fig. 3c), clearly implicating starch metabolism by *B. thetaiotaomicro*n in this interaction.

What molecular cross-feeding interactions drive this interaction? Starch breakdown by sus proteins initially proceeds extracellularly^59^, potentially releasing common goods. The proteomics data indicates downregulation of PTS sugar transporters and upper Embden–Meyerhof–Parnas/glycolysis proteins in the presence of many partner species, and strong upregulation in enzymes involved in butyrate production (Fig. 3d), consistent with previously described cross-feeding of succinate^60^ and acetate^19^ from *Bacteroides* to *Clostridia*. Related to this, the pH of *C. difficile*–*Bacteroides* co-cultures is acidic, potentially alleviating the self-inhibiting effects of the high pH observed in *Clostridium* mono-cultures. Overall, this illustrates the value of proteomic data for generating hypotheses about exchanged metabolites and we next set out to further investigate interactions at the metabolomic level.

### Targeted metabolomics reveals emergent metabolism and cross-fed metabolites

**Fig. 4:**
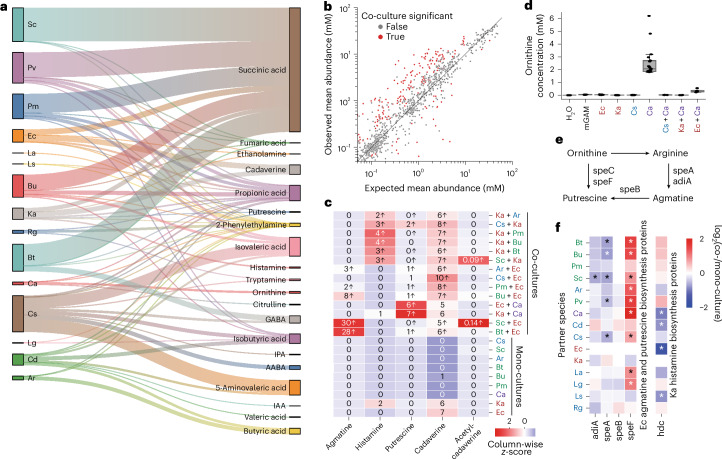
Polyamine and SCFA metabolism are shaped by cross-species interactions. **a**, Metabolites produced individually by gut bacteria in mono-culture. The bar width is proportional to concentration. **b**, A comparison of observed and expected metabolite concentrations in co-cultures. Each dot represents a metabolite in a specific co-culture. Expected concentrations were computed from mono-culture concentrations and the abundance of species in co-culture. Observed concentrations deviating significantly from expected are shown in orange (*P*_adj_ < 0.05, FDR-corrected two-sided Welch’s *t*-test, log_2_(abs(fold change)) >0.5 and minimum observed or predicted concentration >50 µM or >500 µM for SCFA). The line represents the expected *x*–*y* relationship. **c**, The emergent polyamine metabolism in selected co-cultures involving *E. coli* and *K. aerofaciens*. Heat map colours reflect column-wise *z*-scores, and numbers reflect the mean concentration in micromoles per litre. Arrows indicate significant increase from the expected value. *E. coli* does not produce agmatine but does so in co-cultures with specific Bacteroidetes. Histamine production by *K. aerogenes* is stimulated by *C. aerofaciens* and *S. copri*, while putrescine is produced in only co-cultures of *E. coli* or *K. aerogenes* with *C. aerofaciens*. Low levels of acetylated cadaverine emerged in co-culture with *S. copri*. **d**, Ornithine accumulates in *C. aerofaciens* mono-cultures but is depleted in co-cultures with *C. sporogenes*, *K. aerogenes* and *E. coli*, possibly indicating cross-feeding (*n* ≈ 4 biological replicates for co-cultures and *n* ≈ 16 biological replicates for mono-cultures). Box plot elements are defined as: centre line, median; box limits, upper and lower quartiles; whiskers, 1.5× interquartile range. **e**, The genes and metabolites involved in agmatine and putrescine synthesis in *E. coli*. **f**, The abundances of enzymes involved in amine synthesis by species. Since ornithine can be metabolized to putrescine via ornithine decarboxylases, ornithine is produced by *C. aerofaciens* but depleted in co-culture with *E. coli*/*K. aerogenes*, it is highly likely that cross-fed ornithine is further metabolized to putrescine in these cases. Abundances of enzymes involved in amine synthesis cannot explain the detected concentrations of agmatine, putrescine and histidine. Asterisks indicate hits. IPA, indole-3-propionic acid; IAA, indole-acetic acid; GABA, γ-aminobutyric acid; AABA, α-aminobutyric acid.

To further the insights into co-culture metabolism, we applied a panel of targeted metabolomic assays (Supplementary Tables 6 and 7) to the co-culture supernatants, covering amino acids, short- and branched-chain fatty acids, tryptophan-derived metabolites and biogenic amines. Comparing metabolite concentrations with fresh mGAM medium, we identified 74 instances where at least one of the tested metabolites was produced by a species in mono-culture (Fig. 4a and Supplementary Table 8; Methods). As expected, Bacteroidetes produced large amounts of the fermentation product succinate. Many species produced the SCFA propionic acid, as well as the branched-chain fatty acids (BCFAs) isobutyric and isovaleric acid from valine and leucine. In addition, we recapitulated well-known specific metabolic activities, such as indole-3-propionic acid (IPA) produced by *C. sporogenes*^61^, polyamines by *E. coli*^62^ and histamine by *K. aerogenes*^63^. These data are complementary to previous surveys of supernatant metabolomics in gut bacteria^64^ as we have used a different growth medium and cover some additional compounds such as SCFA and BCFA.

Next, we compared metabolite abundances in co-cultures to expected values on the basis of mono-culture concentrations and relative species abundances under an additive model (Fig. 4b; Methods). We identified 267 out of 2,600 instances where a metabolite was differentially abundant in a given species pair (*P*_adj_ < 0.05, FDR-corrected two-sided Welch’s *t*-test, log_2_(abs(fold change)) >0.5 and minimum observed or expected concentration >50 µM). In 73 cases, the observed concentrations were below expected, which indicates either competition for precursor metabolites or the partner species consuming the produced metabolite (cross-feeding). In the remaining 194 cases, concentrations were higher than expected, indicating upregulation of metabolic activity due to species–species interactions, resource partitioning among co-culture members or emergent metabolism.

**Extended Data Fig. 7:**
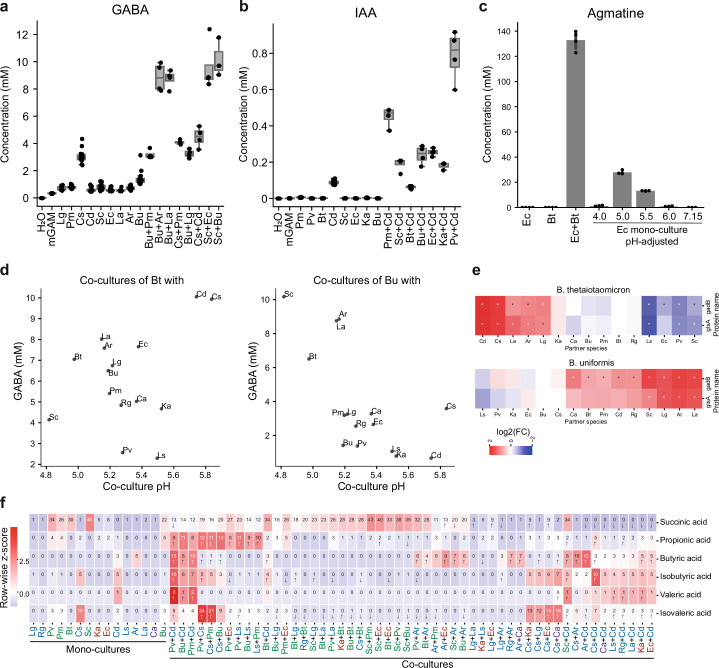
Additional metabolomics analyses. (**a**) γ-Aminobutyric acid (GABA) concentrations from main metabolomics dataset (n ≈ 4 biological replicates per co-culture) for emergent co-cultures and relevant mono-culture conditions. (**b**) Indole-acetic acid concentrations from main metabolomics dataset (n ≈ 4 biological replicates per co-culture) for emergent co-cultures and relevant mono-culture conditions. (**c**) Agmatine concentration in supernatant in an independent small-scale experiment testing the effect of co-culturing (n = 4 biological replicates) and pH (n = 3 biological replicates). Bar heights represent the mean. Media pH was adjusted to the indicated value using hydrochloric acid. *E. coli* growth was substantially inhibited at pH 4, explaining the absence of agmatine. (**d**) GABA concentrations in co-cultures involving *B. thetaiotaomicron* (left) and *B. uniformis* (right), depending on co-culture pH. *B. uniformis* cultures show increases GABA concentrations with decreasing pH, while no such relationship is observed for *B. thetaiotaomicron* co-cultures over a similar pH range. (**e**) Regulation of GAD operon genes recapitulates observed changes in GABA concentrations, including an upregulation in *B. thetaiotaomicron* in co-culture with *C. difficile* and and *C. sporogenes*. Protein identifiers in *B. thetaiotaomicron*: glsA - Q8A4M8; gadB - Q8A4M9. Protein identifiers in *B. uniformis*: glsA - UPI0006C4376F; gadB - UPI00015BEEBA. Dots represent the mean of n = 3 biological replicates, errorbars indicate the standard deviation. The solid line represents the expected x = y relationship. (**f**) Cross-species interactions shape distribution of fermentation end products. Heatmap colours indicate row-wise z-scores. Numbers in the heatmap indicate the concentration in mM. Arrows indicate significant deviation from the expected value in the indicated direction. Many Bacteroidetes produce large amounts of succinic and propionic acid and this is not additive in co-culture indicating competition for precursors. Several species can stimulate butyric acid production by *C. difficile* and *C. sporogenes*. Isobutyric and isovaleric acid production is suppressed in several co-cultures potentially indicating competition for the amino acid precursors. Following convention, box-plot elements are defined as follows: centre line, median; box limits, upper and lower quartiles; whiskers, 1.5× interquartile range.

Among the emergent metabolites is γ-aminobutyric acid (GABA), a neurotransmitter and key effector in the gut–brain axis^65^. Although only produced at low levels in mono-cultures, it emerged as an abundant metabolite in a set of co-cultures (Extended Data Fig. 7a,d,e), many of them involving *B. uniformis*, a known GABA producer^66^. Similarly, levels of indole-acetic acid (IAA), a key microbial metabolite affecting the immune system^26^ and cancer therapy success^67^, were boosted in several co-cultures involving *C. difficile* (Extended Data Fig. 7b). This observation is supported by recent findings that cross-feeding interactions are key to producing health-relevant indole metabolites^68,69^.

Notable emergence was also detected for biogenic amines, including polyamines, which are produced from amino acid precursors, with important consequences for the host^62,63,70^ (Fig. 4c). Agmatine emerged in co-cultures of *E. coli* with *B. uniformis*, *S. copri*, *Parabacteroides merdae*, *A. rectalis* and *B. thetaiotaomicron*. Agmatine production is known to be induced by acid stress^71–73^, consistent with media acidification by Bacteroidetes (Extended Data Figs. 4b and 7c). Here, putrescine emerged in co-cultures of *C. aerofaciens* with *E. coli* and *K. aerogenes*, and the same conditions showed evidence for cross-feeding of ornithine produced by *C. aerofaciens* (Fig. 4d). Ornithine is converted to putrescine by decarboxylation (Fig. 4e), and it is therefore likely that the emergence of putrescine is due to cross-feeding of its precursor. Furthermore, histamine production by *K. aerogenes* was boosted above baseline by a set of partner species including *B. thetaiotaomicron* and *S. copri*. Cadaverine was produced by both *E. coli* and *K. aerogenes*, but this baseline production was not modulated in any co-culture. However, co-cultures with *S. copri* contained acetyl-cadaverine, suggesting that *S. copri* modifies cadaverine produced by *E. coli* and *K. aerogenes*. Acetylation neutralizes the positive charge of polyamines with potential effects on bacterial physiology^74,75^.

We next investigated if the changes in amine production can be explained by changes in enzyme abundance. We Identified no strong expression changes that correlate with the observed increases in amine production (Fig. 4f), indicating that these phenomena originate from the post-translational or metabolic level. A notable exception is the ~9-fold upregulation of *E. coli* SpeF in the presence of *C. aerofaciens*, which correlated with an increase in putrescine in the co-culture. However, multiple other co-culture partners caused a similar (albeit weaker) upregulation of SpeF without concurrent putrescine production (Fig. 4e,f). These results suggest that amine metabolism in the gut is emergent and highly dependent on the nutritional environment. Although species such as *E. coli* and *K. aerogenes* clearly possess the genetic repertoire to produce a range of amines at high concentrations, this does not happen in all conditions, even if the precursors are abundant.

We also noted numerous interactions involving fermentation end products such as succinate, SCFA and BCFA (Extended Data Fig. 7f). Propionic acid was depleted in several co-cultures, but also surprisingly emerged in a small number of cases, three of which involved *L. saccharolytica* and a Bacteroidete. Succinate was depleted and butyrate emerged in several co-cultures of *Clostridia* with *Bacteroides*, in accordance with proteomic changes coherent with acetate and succinate cross-feeding (Fig. 3d). Overall, these results underline the emergent and complex nature of communal metabolism and provide a framework for identifying potential metabolic interactions through comparative metabolomics.

### Narrow amino acid preferences indicate niche partitioning

Gut bacteria obtain amino acids from proteins/peptides in partially digested food^76^ and from host-produced mucin^77^, which in turn impact host amino acid status^78^. Many gut bacteria secrete proteases to digest peptides in the environment, thereby releasing a mix of amino acids^79,80^, yet gut bacteria were found to often have a narrow preference for amino acids^78^. Furthermore, amino acid auxotrophies are common among gut bacteria^81,82^ and amino acids have been proposed as prebiotics^83^. Still, substantial knowledge gaps remain around communal metabolism of amino acids. We therefore next focused on the metabolism of proteinogenic amino acids.

**Fig. 5:**
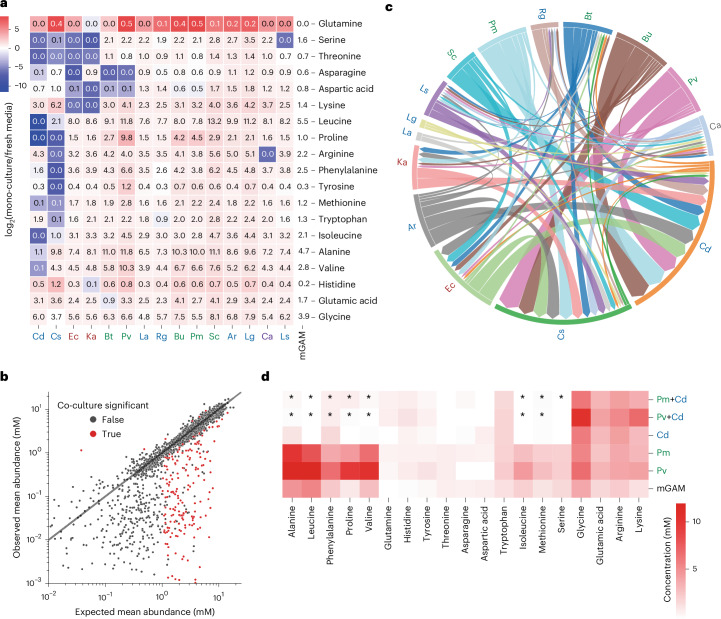
Narrow amino acid preferences result in niche partitioning and cooperative protein digestion. **a**, A heat map illustrating the concentrations of free amino acids in mono-cultures versus fresh mGAM medium. Colours indicate the log_2_-transformed fold change. The numbers in the heat map indicate mean concentrations in millimoles per litre (*n* ≈ 16 biological replicates). **b**, A comparison of expected amino acid concentrations based on mono-cultures, relative abundances and co-culture ODs, with those observed in the co-cultures. Colours denote statistically significant interactions (abs(log_2_(fold change)) >0.5, minimum predicted or observed concentration >1 mM). The line represents the expected *x*–*y* relationship. **c**, The amino acid exchange network reconstructed using the data on amino acids unexpectedly depleted in co-cultures (identified in **b**), where donor and recipient species could be assigned (Methods). Ribbon widths reflect the number of exchanged amino acids. **d**, A heat map illustrating amino acid concentrations across mono- and co-cultures involving *C. difficile* and different Bacteroidetes (*S. copri*, *P. merdae* and *P. vulgatus*). Asterisks denote significant interactions (refer to **b**). The accumulation of a group of amino acids in the Bacteroidetes mono-culture compared with fresh mGAM medium, but their absence in both *C. difficile* mono-culture and co-cultures, indicates that these are probably consumed by *C. difficile*.

We determined the concentrations of free amino acids in the supernatant of our 608 co-culture and mono-culture samples. We found that mono-cultures of most species were depleted in none or very few amino acids (Fig. 5a). While the uptake and intra-cellular metabolism of small peptides is not captured by these data, it is apparent that, in terms of free amino acids, gut bacteria have a narrow preference/requirement for amino acids. The closely related species *E. coli* and *K. aerogenes* had a similar preference profile and depleted lysine, serine, threonine and aspartate. However, the two closely related species *C. difficile* and *C. sporogenes* had distinct and broad profiles overlapping in serine and threonine only. Other species had notably narrow and distinct preference profiles, with, for example, *C. aerofaciens* selectively depleting arginine and *L. saccharolytica* depleting serine.

This pattern could indicate niche partitioning in the gut community, in a scenario where extracellular proteins/peptides are digested collectively and specific released amino acids are used by different species. To investigate this, we compared observed amino acid concentrations in the co-cultures to expected values on the basis of the absolute abundance of each species and the observed consumption/release pattern observed in mono-culture (Fig. 5b). Out of 1,976 potential amino acid–species pair interactions, we detected 187 cases where the observed concentrations differed from the expected (*P*_adj_ < 0.05, FDR-corrected two-sided Welch’s *t*-test, abs(log_2_(fold change)) >0.5, minimum predicted or observed concentration of 1 mM). In all but six cases, the amino acid decreased, suggesting that one of the species is consuming amino acids released by the other.

In 156 cases, we could predict the direction of amino acid flow on the basis of the consumption/release patterns in the mono-culture, resulting in the metabolite exchange network shown in Fig. 5c. *K. aerogenes*, *E. coli*, *C. sporogenes*, *C. difficile* and *B. thetaiotaomicron* were overall receivers of amino acids. Serine, arginine and leucine stood out as being exchanged the most (30, 17 and 16 pairs, respectively). Notably, the opportunistic pathogen *C. difficile*, with a relatively broad amino acid uptake pattern, received amino acids from a range of Bacteroidetes (Fig. 5d). Our data thus show that amino acids obtained by proteolysis are divided amongst co-culture members with narrow-range requirements indicating both niche partitioning and nutrient sharing.

### Regulation of orthologous genes is highly divergent

Our analyses revealed complex interactions at the level of growth, protein expression and metabolism. We next set out to leverage the data for functional and comparative genomics. Taking advantage of the evolutionary diversity of the dataset, we investigated the extent to which the regulatory responses of orthologues to partner species are conserved.

**Fig. 6:**
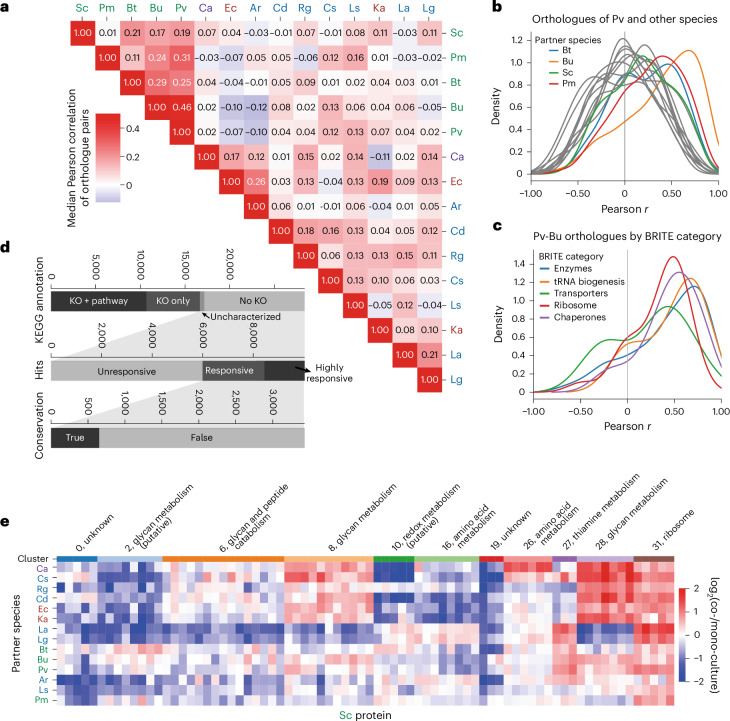
Evolutionary divergence of proteome responses and functional genomics. **a**, The regulation of orthologues in response to other species is conserved only amongst closely related species. Median Pearson correlation coefficients between orthologue pairs of species pairs. The analysis was restricted to single-protein orthologue pairs, reliably detected proteins (<4 missing) and strongly changing proteins (sum(abs(log_2_(fold change))) >2). **b**, The distributions of Pearson correlation coefficients of orthologue pairs between *P. vulgatus* and other species. Only closely related Bacteroidetes species (highlighted in colours) show a shift towards positively correlated expression profiles, indicating similar expression patterns. **c**, The different functional categories of proteins displayed different levels of conservation in their expression profiles. Enzymes and transporters were the most similar, while ribosomal proteins were less similar and transcription factors displayed a broader distribution. **d**, From top to bottom: barplot illustrating the status of KEGG annotation across the dataset, responsiveness of proteins with uncharacterized KOs or without an assigned KO term, and conservation for responsive and common responsive proteins without a KO term. Conservation was defined as eggNOG OG present in >70% of bacterial species. This highlights the very large number of responsive and common responsive proteins without any functional annotation. The approximately 1,800 proteins therein, with strong conservation across bacteria, are priority unstudied genes in the context of communal gene expression. **e**, A clustered heat map showing a selection of highly regulated *S. copri* proteins. Several clusters are made up of proteins with similar functions indicating that co-expression clustering can inform protein function. Many clusters contain only genes with no existing functional annotations. Hierarchical clustering was performed using the Euclidean distance metric and the Ward method. The colour scale was clipped at −2, 2. The numbers at the top indicate the cluster ID (see Supplementary Tables 9 and 10 for all clustering results). Source data

We used Orthofinder^84^ to identify pairs of orthologous proteins and computed Pearson correlation between their abundance changes across co-culture conditions. In the cases of distantly related species pairs, we observed virtually no correlation between orthologue expression profiles (Fig. 6a). Only closely related species showed notable correlation, for example, *E. coli*–*K. aerogenes* (*r* = 0.19), *L. acidophilus*–*L. gasseri* (*r* = 0.21) and *Bacteroides* pairs. Exemplarily focusing on the species *P. vulgatus*, orthologue pairs with *B. uniformis* and *B. thetaiotaomicron* exhibited median correlations of 0.46 and 0.25, respectively (Fig. 6b). The *B. uniformis*–*P. vulgatus* correlation was unexpectedly strong since the more closely related *B. uniformis*–*B. thetaiotaomicron* pair showed a weaker correlation. For the former pair, we noted distinct correlation patterns for different classes of proteins, with enzymes and transporters showing the tightest and weakest correlations, respectively (Fig. 6c).

Although some studies have compared expression profiles of orthologous genes at the transcriptome level, for example, refs. ^85–87^, the evolution of protein abundances remains understudied. Our results indicate that the condition-specific regulation of protein expression is highly variable and even closely related species execute different expression profiles in response to the same partner species. This could point to both functional diversification at the protein level (homologous proteins performing different functions and therefore being regulated differently) and at the species level (species reacting differently to a similar environment as it affects them differently owing to metabolic or cellular differences). The divergent regulation of orthologous proteins in gut bacteria thus highlights the need to account for interspecies interactions in understanding protein and organismal functions.

### Poorly characterized proteins are responsive in co-culture and cluster according to function

The interpretation of gene expression data depends on the availability and quality of functional annotation of proteins. However, a large fraction of bacterial genes, even in relatively well-studied gut bacterial species, remains functionally unannotated. In our set of species, 52% of proteins in the genome and 35% of the measured proteome were not annotated to a KEGG Orthology (KO) term or were annotated to an uncharacterized KO (Fig. 6d). This ‘genetic dark matter’ presents an enormous blind spot in omics studies^1,88,89^. Overall, 34% of these poorly characterized proteins were responsive (at least one hit) or common responsive (at least four hits) in our dataset. Out of the poorly annotated but responsive proteins, 21% (*n* = 652) were conserved across bacteria (data from eggNOG; Methods), indicating a key physiological role in the community context. This approach therefore enables the prioritization of unstudied genes for further, focused experiments.

We next used an unsupervised clustering approach to group proteins with similar expression profiles. Out of 473 clusters across all species, 230 (49%) were enriched in at least one KEGG pathway, and 362 clusters (77%) contained at least one gene pair that shared a genomic neighbourhood. These findings indicate that this approach can identify groups of proteins with related biological functions.

**Extended Data Fig. 8:**
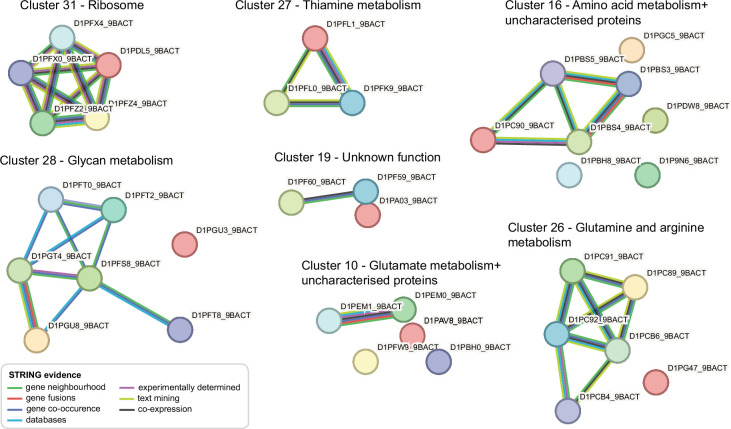
StringDB networks of S. copri clusters. Selected clusters of *S. copri* proteins (Fig. 6e). Networks illustrating known connections between cluster members based on various types of data curated in StringDB.

To explore the use of systematic co-culture proteomics for functional genomics, we focused on *S. copri*, an abundant yet understudied gut microbe negatively correlated with Western diet^90^. From 44 clusters in total, we selected a subset of smaller clusters with highly coherent and diverse expression profiles (Fig. 6e and Extended Data Fig. 8). Some of these contained genes clearly attributable to specific functions, such as cluster 26 which contained arginine biosynthesis genes upregulated in co-culture with *C. aerofaciens*, cluster 27 which contained thiamine biosynthesis genes upregulated in a range of co-cultures, or cluster 31 which contained ribosomal proteins. In some clusters, annotated proteins clustered with uncharacterized ones, suggesting shared function. For example, cluster 28 contained several poorly characterized carbohydrate metabolism genes together with xylan- and sialate-metabolizing enzymes in the same genomic neighbourhood. However, many clusters showed no existing high-quality functional annotation for any proteins. For example, cluster 19 contains three neighbouring genes and was strongly downregulated in co-culture with *C. sporogenes* and others. This case study illustrates the potential of using systematic (meta-)proteomics for co-expression analysis and functional genomics. Full information on clusters across all species is available in Supplementary Tables 9 and 10. An interactive shiny app to explore the clustered expression profiles of all species is available via shiny apps at https://stephan-kamrad.shinyapps.io/co-culture_proteomes/.

## Discussion

A key aspect of community ecology is the emergence of collective functions. Our systematic study enabled expansion of the cases of emergent metabolism previously observed in gut bacterial communities, for example, in the case of bile acids^91^ or carbohydrate metabolism^19^. Our findings include sequential metabolism, for example, acetyl-cadaverine production in *E. coli*–*S. copri* co-cultures, and activation of a secondary metabolic pathway, for example, the stimulation of agmatine in *E. coli* by some Bacteroidetes. We also identify candidate cross-fed metabolites as those depleted in co-cultures of species with complementary production–consumption profiles, for example, ornithine being specifically depleted in *C. aerofaciens*–*K. aerogenes* co-cultures. Together, we demonstrate that metabolomics of combinatorial co-cultures is a promising strategy for identifying metabolic interactions and cometabolism.

The observed metabolic interactions are closely intertwined with the proteome, evident in the strong enrichment of enzymes and transporters among the responsive proteins. Some metabolic changes correlated directly with enzyme abundances, for example, GABA synthesis in *Bacteroides* and arginine biosynthesis in response to the arginine consumer *C. aerofaciens*. In other cases, metabolic changes appear to occur without changes in enzyme abundance, for example, polyamine synthesis. This is congruent with pathways being regulated by either enzyme availability (enzyme control) or by metabolite availability (metabolic control). Thus, the proteome and metabolome serve as complementary readouts, with enzyme regulation data indicative of metabolic changes directed by the cellular regulatory machinery. In the case of *C. difficile* interactions with *Bacteroides*, our comprehensive dataset captures interactions relating to starch metabolism, SCFA and succinate cross-feeding, sharing of amino acids and modulation of pH. This multiplicity of interactions, each depending on nutrient availability/media composition, explains the diversity of interaction types observed in these species in literature^14,55–58,92^. Our results thus exemplify how the functional manifestation of the genetic repertoire is plastic and dependent on the community context.

Omics of in vitro cultures have resulted in deep insights into the biology of a few model bacteria such as *E. coli* and *Bacillus subtilis*. However, many abundant and prevalent species remain massively understudied. Beyond relatively well annotated protein classes such as enzymes, the lack of functional annotation represents a major hurdle to the interpretation of omics data from non-model bacterial species and communities including them. In our dataset, more than a third of genes were not annotated to a KO term. If large proteomic datasets are available, covariation analysis can be a powerful way of gene functional annotation, which at least in human^93^ and yeast^94^, outperforms other strategies of functional annotation of unknown protein function, and is a particularly attractive strategy for annotating gene function in species that are difficult to manipulate genetically. We have here shown that systematic co-culture proteomics cluster into functionally enriched sets of proteins. At the same time, our case study of *S. copri* shows that many strongly and specifically regulated protein clusters are entirely unannotated to date, thereby identifying priority unstudied genes in the community context. Our co-culture proteomics data is thus a useful complementation to other current systematic functional genomics efforts in the microbiome space, for example, involving forward genetic screens through transposon mutagenesis^95,96^.

Taken together, our data open a molecular window into bacterial interactions with implications for functional genomics and for engineering community metabolism.

## Methods

### Cultivation of bacteria and sample collection

Bacterial isolates were obtained from culture collections (Supplementary Table 1). All cultures were grown statically at 37 °C in an anaerobic polyvinyl chamber (Coy Instruments) filled with 2.5% H_2_, 12% CO_2_ and balance N_2_. Cultures were grown in mGAM, produced by Nissui Pharmaceuticals and obtained from HyServe, prepared according to the instructions from the manufacturer and sterilized by autoclaving. From glycerol-preserved cryostocks, 10 ml cultures were grown in screw-top tubes for 1 or 2 days. Cultures were then diluted 100-fold into 10 ml of fresh media and incubated again for 1 day. The OD at 600 nm of each culture was measured and a dilution was prepared. Using a 96-channel pipetting robot, diluted cultures were combined in all possible pairs in 96-well deep-well plates at an initial OD of 0.05 and a volume of 1.4 ml. Columns 9–12 of each plate were used for mono-culture controls, which had the same volume and initial OD. Plates were sealed with a breathable film and incubated for 24 h.

Plates were then removed from the anaerobic chamber, cultures were mixed well and 100 μl was transferred into a fresh 96-well plate, which was used to measure the absorbance at 595 nm (OD) with a microplate reader (note that the resulting values are not directly comparable to OD values obtained with a standard spectrophotometer). The remaining cultures in the deep-well plates were then centrifuged (5 min, 3,200*g*) and three aliquots (80 μl each) of supernatant were collected for metabolomic analysis. The rest of the supernatant was discarded and 1 ml of water was added to wash off residual media from the cell pellet. Plates were shaken briefly before centrifuging again with the same settings. The supernatant was discarded and cell pellets frozen at −80 °C until processing for proteomics.

### Proteomics sample preparation

Proteomics samples were prepared on the basis of a previously established high-throughput protocol^94^, with a few key modifications. An aqueous lysis buffer with the following composition was freshly prepared: 7 M urea, 100 mM ammonium bicarbonate, 24 mM Tris–HCl, 2.4 mM MgCl_2_ and 122 units ml^−1^ benzonase (Sigma-Aldrich). A small amount of glass beads and 200 μl of lysis buffer were added to each pellet and the plates were sealed with rubber mats. Cells were lysed using beat beating (2× 5 min, 1,500 rpm), followed by a brief centrifugation and incubation for 20 min at 37 °C in a water bath (for benzonase digestion). For reduction-alkylation, 20 μl of 55 mM dithiothreitol was added, plates were mixed briefly and incubated at 30 °C for 1 h, followed by 20 μl of 120 mM iodoacetamide, brief mixing and 30 min at room temperature in the dark. Then, 450 μl of 0.1 M ammonium bicarbonate was added, plates were mixed briefly and centrifuged for 5 min at 3,220*g* to clear the extract from cell debris.

Using an automated workflow on a liquid handling robot and the previously obtained OD measurements of each culture, 0.72 OD units of extract were transferred to a fresh deep-well plate and adjusted to a final volume of 500 μl using an aqueous solution of 2 M urea and 0.1 M ammonium bicarbonate. This ensured an approximately even input biomass for all samples, while minimizing the time that live bacterial cells are handled aerobically before lysis and denaturation.

For digestion, 10 μl of trypsin/LysC mix (Promega, cat. no. V5072, prepared according to instructions from the manufacturer) was added to each sample, with subsequent incubation at 37 °C with shaking for 17 h. Digestion was stopped by adding 25 μl of 25% formic acid. Solid phase extraction in a 96-well format was used to clean up the samples. SPE plates (BioPureSPE PROTO C18 MACRO, Nest Group, cat. no. HNS S18V-L) were first conditioned with methanol, followed by buffer A (50% acetonitrile in water) twice and buffer B (water with 0.1% formic acid) twice, using a volume of 200 μl per well throughout. The entire digest was then loaded and washed three times with buffer B before eluting with 110 μl of buffer A three times. Samples were dried in a concentrator (Savant SPD300DDA) at 35 °C for approximately 6 h. Samples were reconstituted in 40 μl of buffer B and peptide concentrations were determined using a fluorometric assay (Pierce Quantitative Fluorometric Peptide Assay, ThermoFisher, used according to manufacturer’s instructions).

### Proteomics data acquisition

Liquid chromatography–mass spectrometry (LC–MS) analysis was performed on an Evosep One system coupled to a Bruker timsTOF Pro 1 mass spectrometer. In total, 200 ng of peptides was loaded on Evotip Pure tips according to the manufacturer’s protocol. Liquid chromatography was carried out using the Evosep 100 SPD LC method (11.5-min gradient) with an EV1109 performance column (ReproSil Saphir C18, 8 cm × 150 µm, 1.5 µm beads by Dr Maisch) at 40 °C, coupled to a 10-µm ZeroDeadVolume captive spray emitter. Data were collected over an *m*/*z* range of 100–1,700 for mass spectrometry on the timsTOF Pro instrument using an accumulation and ramp time of 100 ms. The ion mobility range was set 0.85–1.27 Vs cm^−2^. DIA-PASEF scans used a cycle time of 0.95 s. Mobility windows included 21 mass steps per cycle with a 25 Da mass width. The collision energy was decreased as a function of the ion mobility from 20 eV (1/*K*_0_ of 60 Vs cm^−2^) to 59 eV (1/*K*_0_ of 1.6 Vs cm^−2^).

### Proteomics data analysis

Proteomics raw data were analysed in two stages using DIA-NN v1.8.2 beta 27 (refs. ^46,97^). In stage 1, all samples were searched against an in silico predicted library generated from the proteome fasta files of all species (obtained from Uniprot^98^) and a fasta file of common contaminants (crap.fasta, https://www.thegpm.org/crap/). DIA-NN was called from the command line using the following options: --qvalue 0.01 --matrices --gen-spec-lib --predictor --fasta-search --min-fr-mz 200 --max-fr-mz 1800 --met-excision --cut K*,R* --missed-cleavages 1 --min-pep-len 7 --max-pep-len 30 --min-pr-mz 300 --max-pr-mz 1800 --min-pr-charge 2 --max-pr-charge 3 --unimod4 --window 18 --mass-acc 15 --mass-acc-ms1 15 --smart-profiling --pg-level 1.

Data from stage 1 were used to assess sample quality. Out of 768 samples, 106 samples were excluded because they had fewer than 4,000 precursor identifications or more than 10% of identified precursors originated from species other than those expected in the sample. We furthermore excluded all remaining samples (*n* = 46) containing the species *Bifidobacterium longum* subspecies *longum* as very few precursors from this species were detected across the dataset, most probably because of the low growth rate of this species and a contamination of the pre-culture. Another eight samples were excluded as these showed abnormally high ODs compared with the other replicates, probably indicating contamination. A total of 608 samples were included for further analysis.

In stage 2, one DIA-NN call was performed per species, searching all samples containing the species against a predicted spectral library for that species only. That is, each co-culture sample was analysed in two separate DIA-NN calls, one for each species. Options were as above, except that we enabled ‘Match Between Runs’, which performs a two-pass search, the second of which with a subsetted library of precursors detected in at least one sample. Peptide reports in parquet format were then further processed in Python and R by removing precursors with Q.Value >0.01, Global.Q.Value >0.01, Quantity.Quality <0.85 and Precursor.Quantity <10,000. Precursors not identified in mono-culture were removed. Non-proteotypic peptides were removed (including those matching to more than one protein within the species, as well as those matching to a protein from another species in the sample). We also removed a small number of peptides which were detected consistently (in more than three cases) in the mono-cultures of the other species in the sample. Finally, precursors were excluded if their CV across the mono-culture replicates was greater than 0.5, indicating strong technical or biological noise.

After precursor filtering, the normalized quantUMS^99^ precursor quantities computed by DIA-NN (column ‘Precursor.Normalised’) were log_2_-transformed and renormalized using cyclic loess normalization as implemented in limma^100^ using default options. Protein quantities were computed with the maxLFQ algorithm^101^ as implemented in the DIA-NN R package after transforming back to normal (that is, not log_2_) scale. A small number of proteins with a CV >0.5 across the mono-culture replicates were excluded. Protein-level summary statistics were computed and statistical analysis was performed with limma by fitting a linear model to the data (using the lmFit function), defining contrasts as each co-culture versus the mono-culture, and using empirical Bayes to obtain moderated *t*-statistics where standard errors were moderated towards a trend using eBayes(trend=True). A protein was considered quantified in a given co-culture condition if it was measured in at least two replicates in co-culture and six replicates in mono-cultures, with at least two matching peptides. Quantified proteins with *P*_adj_ < 0.05 and abs(log_2_(fold change)) >0.5 were considered hits.

### Determination of species abundances

We used the peptide report from stage 1 (above), filtered for proteotypic peptides (across all species) and *q* value and Quantity.Quality filters as before. For each species, highly complete precursors were selected (identified in >90% of samples) and missing values imputed using median imputation. The sum of non-normalized precursor quantities (column Precursor.Quantity) was used as proxy of relative species abundance, which was divided by the same value obtained for the mono-culture of that species to correct for the fact that an identical amount of injected peptides will produce different intensity sums for each species (for example, owing to the quality of the annotated proteome or physicochemical peptide properties). For each co-culture sample, the quantities obtained for each species were divided by the sum of both to obtain relative abundances for each sample. These were then scaled by the total OD_600_ of the co-culture to obtain absolute abundances of each species in the co-culture in OD units.

### Benchmark experiment

To test for potential artefacts introduced by matrix effects, mono- and co-cultures of *C. difficile*, *B. thetaiotaomicron* and *A. rectalis* were grown in 50 ml mGAM under otherwise identical conditions to those described above. Cells were pelleted (3,200*g*, 20 min, 4 °C) and resuspended in the required volume of water to obtain a cell suspension with an OD of 7.2. A volume corresponding to 1.5× of the mono-culture OD units (1.25 for *C. difficile*, 3.4 for *A. rectalis* and 5.3 for *B. thetaiotaomicron*) was aliquoted in quadruplicates into a 96-deep-well plate (this mirrors the original screen which used 1.5 ml culture volume). Co-cultures were treated in the same way corresponding to the co-culture OD (aliquoting 4.2 OD units for *C. difficile* and *A. rectalis* and 5.1 OD units for *A. rectalis* and *B. thetaiotaomicron*). Co-extraction controls were prepared by mixing mono-culture cell suspensions in the appropriate ratio (derived from the relative abundance observed in the main screen, 3:7 for *C. difficile*:*A. rectalis* and 9:1 for *A. rectalis*:*B. thetaiotaomicron*). The plate was then centrifuged to collect the cells and stored at −80 °C. Samples were then prepared as described above. After sample preparation, a pool of mono-culture samples was used to create the comeasurement samples, by mixing samples in the same ratio as stated above. Data were acquired on the same instrument and method as for the main experiment.

### pH measurements

High throughput colourimetric pH estimations were conducted as described previously^102^, with slight modifications. In brief, a 21-point pH calibration curve between pH 4.03 and 8.08 was prepared in mGAM and the pH of each calibrant was measured using a micro pH meter (ThermoScientific 8220BNWP). Then, 10 μl of 10 mg ml^−1^ litmus dye was added to 50 μl of each calibrant, as well as supernatant samples, in half-area flat-bottom plates (Greiner, cat. no. 675801). Full absorption spectra (350–800 nm with a 5 nm step-size) of three replicates of each point in the calibration curve were acquired on a Varioskan (ThermoScientific VL0000D0) and used to determine the isosbestic point (the wavelength corresponding to minimum absorption variance across calibrants and the wavelength corresponding to maximum absorbance variance across the calibrants). Absorbance measurements at these two wavelengths (430 nm and 585 nm, respectively) were acquired for all calibrants and test samples. A sigmoidal curve fitted using the absorbance ratio (absorbance at 430 nm:absorbance at 585 nm) and calibrant pH was used to estimate supernatant sample pH.

### Prediction of responsive proteome fraction

The multiple linear regression model was built with scikit-learn. Features were centred and scaled using the scale from sklearn.preprocessing. Linear regression was performed with LinearRegression from the sklearn.linear_model. Cross-validation was performed using LeaveOneOut from the sklearn.model_selection. Default settings were used throughout.

### Metadata and genomic analysis

#### Functional annotation

KEGG annotations for protein sequences were retrieved with the GHOST Koala tools (v2.0) via the web interface. EggNOG annotations for protein sequences were obtained using the eggNOG mapper v2.1.12^103^ via the web interface. Genomes were retrieved from sources listed in Supplementary Table 1 and gene locations were identified using tblastn^104,105^. The conservation of proteins across bacteria was computed using data downloaded from eggNOG (v5.0)^103^. Specifically, the table of proteins assigned to each eggNOG orthologous group (OG) (/download/eggnog_5.0/per_tax_level/2/2_members.tsv.gz) was used to count the number of species in which a given OG is present and this number was divided by the total number of bacteria in the database (5090, see file e5.taxid_info.tsv in the same folder). A protein was considered conserved if it was present in more than 70% of species.

#### Enrichment analyses

Fisher’s exact test as implemented in the scipy.stats module was used to test for significant enrichment. All measured proteins (of the respective species) were used as the background set. Enrichment was quantified as (hit proteins annotated to term/all hit proteins)/(background proteins annotated to term/background proteins).

#### Clustering

Clustering was performed on log_2_-transformed fold-change data (co-/mono-culture). For clustering, proteins with more than three missing values were removed. We also excluded proteins which showed very little response across conditions (sum(abs(log_2_(fold change))) <2). Remaining missing values were imputed with zero. Hierarchical clustering was performed with the Euclidean distance metric and Ward linkage method, as implemented in AgglomerativeClustering from the scikit-learn sklearn.cluster module. The number of clusters was set to the number of proteins divided by 20.

#### Orthologue analysis

Orthologues were identified using OrthoFinder v2.5.5^84^ with default settings using protein sequences as input. To compute distributions of orthologue expression profile correlations, the OrthoFinder output for each species pair was filtered to only include single-protein ortho-groups and only those where proteomics data was available for both proteins. Pearson correlation was used to compute the similarity of two orthologues’ expression profiles across co-culture conditions. Phylogenetic distances between species were computed on the basis of the previously generated tree using the cophenetic.phylo function of the ape R package^106^.

### Quantitative flow cytometry

Cells were stained to distinguish gram-positive and negative cells by flow cytometry^107^. Cells from 1 ml of overnight culture were collected by centrifugation (5 min, 8,000*g*), washed in 1 ml 1 M KCl, resuspended in 400 µl of 4% formaldehyde diluted in 1 M KCl, and incubated on ice for 30 min to fix the cells. The cell suspension was then centrifuged and the pellet was washed once with 1 M KCl. Cells were resuspended in 300 µl of 1 M KCl and stored at 4 °C until analysis. For staining, the samples were first diluted to OD 0.5 in 300 µl of 1 M KCl, followed by the addition of 4 µg ml^−1^ Vancomycin BODIPY FL Conjugate (Invitrogen, cat. no. V34850) and 15 min incubation at 30 °C in the dark. Cells were collected by centrifugation and resuspended in 300 µl of phosphate-buffered saline in polystyrene tubes for flow cytometry.

Samples were run on a BD LSRFortessa cell analyser using the BD FACSDiva software (v9.0.1) with the following settings: slow speed acquisition mode, laser 488 nm and the Blue530, forward scatter (FSC) and side scatter (SSC) filter for acquisition. Fcs files were analysed with FlowJo (v10.9.0) gating the cell population in the FSC-H/SSC-H plot, following a singlet gating on SSC-H/SSC-A plot and gating in the in a Blue530-H/FSC-H dimensions for discriminating gram-positive (*C. difficile*) and gram-negative (Bacteroidetes, *E. coli*) bacterial cells (Extended Data Fig. 3). The same samples were used for cell counting using the bacteria counting kit (Invitrogen, cat. no. B7277) following the manufacturer’s protocol. Fixed cells were diluted to obtain a sufficient signal in the bead gate upon flow cytometry analysis and stained with Sytox so that the cells could be discriminated from the beads using the Blue530/30 and FSC filters (Extended Data Fig. 3).

### *B. thetaiotaomicron* sus mutant

*Escherichia coli* S17λpir was grown in Miller’s lysogeny broth (LB; Corning) supplemented with ampicillin (100 μg ml^−1^) when required and incubated at 37 °C with 180 rpm shaking. Genetic constructions were made in the *B. thetaiotaomicron* VPI-5482Δ*tdk* background, developed for a two-step selection procedure of unmarked gene deletion by allelic exchange^108^. All primers used in this study are listed in Extended Data Fig. 6a and were designed de novo on the basis of the VPI-5482 reference genome using SnapGene to enable Gibson assembly into the suicide vector pLGB13. Allelic exchange primers consisted of a gene-specific region (~25–35 bp) and a 5′ overlapping sequence (15–20 nucleotides), resulting in total primer lengths of ~50–60 bp; vector-linearization primers were ~20 bp. Oligonucleotides were synthesized and supplied in liquid form by Merck (Sigma-Aldrich). Mutants were generated via allelic exchange using the suicide vector pLGB13^109^. Approximately 500 bp regions flanking the target gene were amplified by PCR using Phusion Flash High-Fidelity PCR Master Mix (ThermoFisher Scientific), and assembled with the plasmid backbone using Gibson assembly. The reaction mix, containing ISO buffer, T5 exonuclease, Phusion HF polymerase and Taq DNA ligase, was incubated at 50 °C for 35 min. The assembled plasmid was then introduced into *E. coli* S17 λpir, which served as the conjugation donor. For conjugation, exponentially growing cultures of donor and recipient were mixed at a 2:1 ratio and spotted on BHI medium agar (supplemented with 5 mg l^−1^ hemin, 2 g l^−1^ NaCOH_3_ and 1 g l^−1^ cysteine), followed by overnight incubation at 37 °C under aerobic conditions. The following day, the mix was plated on BHI agar supplemented with erythromycin (15 µg ml^−1^) to select for *B. thetaiotaomicron* transconjugants that had undergone the first recombination, and gentamicin (200 µg ml^−1^) to inhibit donor *E. coli* growth. Resulting colonies were cultured overnight in antibiotic-free BHI medium to facilitate plasmid loss, then plated on BHI agar containing anhydrotetracycline to counterselect against cells retaining the vector. Candidate deletion mutants were verified by colony PCR using flanking primers followed by Sanger sequencing.

Growth on single carbon sources was tested in M9 defined media containing a single carbon source, glucose or amylopectin (a branched polymer of glucose) (Extended Data Fig. 6b). M9 defined medium consisted of M9 salts (6 g l^−1^ Na_2_HPO_4_, 3 g l^−1^ KH_2_PO_4_, 0.5 g l^−1^ NaCl and 1 g l^−1^ NH4Cl), 0.246 g l^−1^ MgSO_4_·7H_2_O, 0.014 g l^−1^ CaCl_2_·2H_2_O, 50 mg l^−1^ cysteine, 5 mg l^−1^ hemin, 2.5 µg l^−1^ vitamin K_3_, 5 µg l^−1^ vitamin B_12_, 2 mg l^−1^ FeSO_4_·7H_2_O and 1 g l^−1^ carbon source (glucose or amylopectin). Amylopectin from maize was autoclaved and dialysed using 3.5 kDa MW membranes before use (Slide-A-Lyzer Dialysis Cassettes, ThermoScientific).

### Metabolomics sample preparation

Supernatant was collected from cultures in a fresh 96-well plate and stored at −80 °C until further processing. For biogenic amine analysis, supernatants were diluted 1:10 in water in a final volume of 80 µl. For amino acid analysis, supernatants were diluted 1:100 in water in a final volume of 80 µl. For all other LC–MS methods, 80 µl of undiluted supernatant was used. (Diluted) supernatants were extracted by protein crash by adding 120 µl of extraction buffer (1:1 acetonitrile:methanol with 0.1% formic and 20 µM each of amoxicillin, caffeine, ibuprofen and donepezil as internal standards), mixing briefly by shaking (15 s, 1,500 rpm), incubating at 4 °C for approximately 30 min and then centrifuging (5 min, 3,200*g*, 4 °C). Then, 15 µl of cleared extract was removed into a fresh 384-well PCR plate for LC–MS analysis.

### Metabolomics data acquisition

All metabolomics measurements were performed on an Agilent Infinity 1290 LC coupled to an Agilent 6470B triple quadrupole mass spectrometer with a JetStream ion source. Multiple reaction monitoring was used to monitor compound-specific precursor–fragment transitions (usually two per compound). A dilution series of a mixed analytical standards, water blanks, media blanks and a quality control sample (obtained from an *E. coli* culture, spiked with standard solution if required) were injected in regular intervals. Samples were injected in a random order. Amino acids were measured using a low-pH hydrophilic interaction chromatography (HILIC) method^110,111^. Short- and branched-chain fatty acids were measured without derivatization on a porous graphitic carbon column (Hypercarb, ThermoFisher)^112^. Organic acids were measured using a high-pH HILIC method^113^. Amines were measured using a shortened low-pH HILIC method adapted from ref. ^114^. Tryptophan metabolites and various other non-polar gut-specific metabolites were measured using a custom reverse-phase method. Further details, including a list of all transitions and method parameters is provided in Supplementary Table 6.

### Metabolomics data analysis

Raw data were analysed using MassHunter Quantitative Analysis (v10.1). Compounds which were not detected in any of the samples or which had bad calibration curves, unacceptably high background signal or bad peak shapes were excluded from further analysis. Concentrations were estimated from peak areas using calibration curves obtained from serially diluted mixed standards. The data were further processed using custom scripts. Only samples matching those used in the proteomics experiment were taken forward for analysis, that is, samples which were removed from the proteomics analysis owing to suspected contamination or other reasons were also removed here.

### Metabolomics statistical analysis

Identification of compounds produced by mono-cultures: To identify which species produces/consumes metabolites in mono-culture, significant differences in the mean concentrations across biological replicates between mono-culture and fresh mGAM were identified using FDR-corrected two-sided Welch’s *t*-test. For proteinogenic amino acids, significance was defined as *P*_adj_ < 0.01 and abs(log_2_(fold change)) >1. For other compounds it was defined as *P*_adj_ < 0.01, fold change >5 and minimum concentration in mono-culture >500 μM for SCFAs and >50 μM for all other compounds.

Interaction analysis: For each sample, metabolite concentrations were subtracted by the mean value obtained for fresh media to obtain concentration changes. For each co-culture sample the expected metabolite concentration was determined by randomly sampling one of the mono-culture concentrations, multiplying by the respective relative species abundance (absolute abundance in co-/mono-culture) and adding these to the baseline concentration in fresh media. An independent, two-sided Student’s *t*-test with FDR correction was used to compare expected to observed concentrations across the approximately four replicates. A significant interaction was called if *P*_adj_ < 0.05, abs(log_2_(fold change)) >0.5 and a minimum observed or predicted concentration >50 µM (1 mM for amino acids). Amino acid donors and recipients (Fig. 5c) were assigned on the basis of the analysis described in the paragraph above.

### Reporting summary

Further information on research design is available in the Nature Portfolio Reporting Summary linked to this article.

## Supplementary information

Reporting Summary

Peer Review File

Supplementary Table 1 Bacterial strains. Supplementary Table 2. Proteomics summary statistics. Supplementary Table 3. Protein metadata. Supplementary Table 4. Ecological interaction types. Supplementary Table 5. Predicting the fraction of responsive proteins. Supplementary Table 6. Metabolomics method details. Supplementary Table 7. Metabolomics summary statistics. Supplementary Table 8. Emergent and cross-fed metabolites. Supplementary Table 9. Gene clusters and functional annotation. Supplementary Table 10. Pathway enrichments of clusters.

## Source data

Source Data for Figs. 1, 2, 3 and 6 and Extended Data Figs. 2 and 5 Proteome statistics.

## Data Availability

The mass spectrometry proteomics data have been deposited to the ProteomeXchange Consortium and are available via the PRIDE partner repository at http://proteomecentral.proteomexchange.org (ref. ^115^) with dataset identifiers PXD055395 (main experiment) and PXD072524 (benchmark experiment). The proteomics data are available via our interactive web app at https://stephan-kamrad.shinyapps.io/co-culture_proteomes/. Targeted metabolomics raw data are available via Mendeley Data at 10.17632/8wsm6tkh6n.1 (ref. ^116^). Proteomics analysis code and intermediate files are available via Mendeley Data at 10.17632/6djkbgs22f.1 (ref. ^117^). Source data are provided with this paper.
