# Supplementary material for: Interspecies interactions drive bacterial proteome reorganization and emergent metabolism
Source: Nat Ecol Evol. 2026 Mar 31;10(6):1127–44. doi: 10.1038/s41559-026-03030-4 (PMC13253324; doi:10.1038/s41559-026-03030-4)
Supplement: Supplementary file 2 — Peer Review File [file 41559_2026_3030_MOESM2_ESM.pdf]

nature portfolio

Peer Review File

Interspecies interactions drive bacterial proteome reorganisation and emergent metabolism

Corresponding Author: Professor Kiran Patil

Version 0:

Decision Letter:

13th August 2025

*Please ensure you delete the link to your author homepage in this e-mail if you wish to forward it to your co-authors.

Dear Professor Patil,

Thanks for your patience while we sought reviewer input for your manuscript entitled "Interspecies interactions drive bacterial proteome reorganisation and emergent metabolism", and apologies for the delay in getting this decision back to you. I can confirm that your manuscript has now been seen by 2 reviewers, whose comments are attached. The reviewers have raised a number of concerns which will need to be addressed before we can offer publication in Nature Ecology & Evolution. We will therefore need to see your responses to the criticisms raised and to some editorial concerns, along with a revised manuscript, before we can reach a final decision regarding publication.

In particular, Reviewer #1 feels that more detail is needed on sample exclusion and statistical thresholds, and they have requested that the data be made available. Reviewer #2 would like to see a clearer discussion of how the work advances the field compared to previous studies.

We therefore invite you to revise your manuscript taking into account all reviewer and editor comments. Please highlight all changes in the manuscript text file.

We are committed to providing a fair and constructive peer-review process. Do not hesitate to contact us if there are specific requests from the reviewers that you believe are technically impossible or unlikely to yield a meaningful outcome.

When revising your manuscript:

* Include a “Response to reviewers” document detailing, point-by-point, how you addressed each reviewer comment. If no action was taken to address a point, you must provide a compelling argument. This response will be sent back to the reviewers along with the revised manuscript.

* If you have not done so already please begin to revise your manuscript so that it conforms to our Article format instructions at http://www.nature.com/natecolevol/info/final-submission. Refer also to any guidelines provided in this letter.

* Include a revised version of any required reporting checklist. It will be available to referees (and, potentially, statisticians) to aid in their evaluation if the manuscript goes back for peer review. A revised checklist is essential for re-review of the paper.

* Extended Data Figures - please ensure that any supplementary figures and tables that are crucial to the manuscript’s conclusions are converted into Extended Data figures and tables to increase visibility of these data. Extended Data figures and tables are online-only (present in the online PDF and full-text HTML versions of the paper), peer-reviewed display items that provide essential background to the article but are not included in the main article due to space constraints. A maximum of ten Extended Data display items (figures and tables) is permitted.

Please use the link below to submit your revised manuscript and related files:

Link Redacted

Note: This URL links to your confidential home page and associated information about manuscripts you may have submitted, or that you are reviewing for us. If you wish to forward this email to co-authors, please delete the link to your

homepage.

We hope to receive your revised manuscript within four to eight weeks. If you cannot send it within this time, please let us know. We will be happy to consider your revision so long as nothing similar has been accepted for publication at Nature Ecology & Evolution or published elsewhere.

Nature Ecology & Evolution is committed to improving transparency in authorship. As part of our efforts in this direction, we are now requesting that all authors identified as ‘corresponding author’ on published papers create and link their Open Researcher and Contributor Identifier (ORCID) with their account on the Manuscript Tracking System (MTS), prior to acceptance. ORCID helps the scientific community achieve unambiguous attribution of all scholarly contributions. You can create and link your ORCID from the home page of the MTS by clicking on ‘Modify my Springer Nature account’. For more information please visit please visit www.springernature.com/orcid.

Please do not hesitate to contact me if you have any questions or would like to discuss these revisions further.

We look forward to seeing the revised manuscript and thank you for the opportunity to review your work.

[redacted]

Reviewer expertise:

Reviewer #1: Microbial systems biology, proteomics

Reviewer #2: Microbial interactions, microbial systems ecology, gut microbiomes

Reviewers' comments:

Reviewer #1 (Remarks to the Author):

The study examined 104 pairwise co-cultures of 15 gut bacteria, revealing diverse ecological interactions—mainly competition, amensalism, and exploitation. It offers important advantages, particularly from a proteomics-informed systems ecology viewpoint, highlighting the molecular scale of ecological interactions that cannot be captured by genomic data alone.
The data and results reported are extensive, and the analysis progresses from broad profiling to deeper mechanistic insights, offering multiple perspectives and novel observations. Overall, it presents a valuable contribution to the field. I do, however, have several comments and suggestions that I believe could further improve the clarity, robustness, and impact of the manuscript.

Major comments
1. Out of 768 samples, 106 were excluded (~14%) is notable and warrants clarification. This is a substantial proportion—can the authors clarify the reasons for such sample loss? Could this be due to competitive supression of growth, technical issues in proteomics processing, or contamination during culturing?
2. Figure 1H is very interesting, as it illustrates how certain species tend to dominate in co-cultures, reflecting their growth advantages—at least under the specific culture medium used in this study. However, this information could be presented more clearly and intuitively. I suggest adding a 14 × 14 matrix of stacked bar plots or pie charts (to replace Figure S1F), where each cell visualizes the relative protein biomass contribution of each species within a given co-culture, similar in style to Figure S1D. This would provide a more immediate and accessible visual impression of species dominance patterns across co-culture conditions.
3. Figure 1K is also interesting, I’ve recently came across a study that discussed co-culture interactions by colony shapes that two species form: https://microbiomejournal.biomedcentral.com/articles/10.1186/s40168-025-02156-0. The authors have concluded a similar stacked column bar chart. It may be worthwhile for the authors to consider comparing and discussing their findings in the context of this study, particularly regarding how different experimental platforms (e.g., colony-based vs. proteomics-based) may reveal similar or complementary interaction patterns.
On an additional note, something I was wondering when reading the above paper: proteomics could be adapted for spatially resolved analysis in plate-based co-culture systems. Given the sensitivity of modern proteomic workflows to low biomass, would it be feasible to perform protein extraction specifically at the colony–colony interface to capture spatial interaction zones? Such spatially resolved metaproteomics could potentially offer unique insights into the localized molecular responses that occur during direct microbial contact.
4. Before mentioning results related Figure 1J-K, the authors should first clearly define how negative an positive interactions, as well as the five different types of ecological interactions – how were these quantified from the dataset? Clarifying this will enhance the reader’s ability to interpret Figure 1J–K, assess the biological relevance of the findings, and understand the ecological implications of the observed patterns. It is also important to clarify whether samples excluded due to poor proteomic quality may have had low protein yield because of underlying ecological factors, such as limited growth or biomass production in specific co-culture conditions. If so, this could introduce bias into the conclusions presented in Figure 1K, as certain interaction types, especially those associated with low-abundance, may be systematically underrepresented.

Were any statistical criteria or thresholds applied in combination with the ecological interaction typing? For example, were detections on changes of biomass supported by significance testing? Clarifying this would strengthen confidence in the robustness and reproducibility of the interaction classifications.
5. The manuscript states that in 1,163 instances (0.25% of the dataset), a protein was only identified in co-culture but not in mono-culture, and these were classified as hits. Could the authors clarify how “only identified in co-culture” was defined? Specifically, was identification required in all technical replicates of the co-culture and absent in all replicates of the mono-culture, or was detection in any single replicate considered sufficient? It is known that in proteomics, even technical replicates of the same sample may yield partially inconsistent identifications. Proper statistical handling and reproducibility filtering are necessary to rule out stochastic detection effects.
6. Supplementary Figure 6, it’s interesting to see GABA responses, wondering if the authors checked the protein levels related to GABA pathways in the co-cultures. Proteins involved in GABA pathways can be annotated using eggNOG, KEGG etc.
7. The response of ‘genetic dark matter’ related proteins (Fig6D) is intriguing and highlights a substantial proportion of co-culture-responsive proteins that remain functionally unannotated. This section would benefit from a more hypothesis-generating exploration of this ‘genetic dark matter’ rather than ending with general statements. For example, it is known that eggNOG annotates proteins not only using KEGG but also other methods such as COG, Pfam , and GO. Are any of these poorly annotated proteins characterized through non-KEGG methods? In addition, some recent studies have attempted to functionally profile microbiome 'dark matter', could any of these approaches be used to further interpret the co-culture responsive yet uncharacterized proteins in this study?
8. The authors should provide reviewer access to the proteomics raw dataset. I wasn’t able to find it in among the submitted files.

Minor comments
1. Line 45, These 45 include abundant and prevalent commensal species from four phyla, better expand what these four phyla are.
2. From a logical perspective, in the first result paragraph of lines 43–52, readers may wonder about the deeper rationale behind the species selection—for example, whether the selection was linked to diverse ecological interactions. Was the choice of species informed by a pre-designed consideration of potential interactions?
3. Line 57, The term “concatenated library-free approach” may be difficult to understand for readers who are not familiar with proteomics. I suggest the authors consider rephrasing it in a more accessible way or briefly explaining the concept upon first mention, to improve clarity and ensure broader comprehension.
4. Line 68, the statement “covers the vast majority of the proteome by mass” lacks direct quantitative evidence from this study. To avoid overstatement, tone down as “is expected to capture the majority of the proteome by mass”.
5. Lines 72-75. The terms “excellent coverage” and “good coverage” for different functional categories (e.g., enzymes, ribosomal proteins, transcription factors) are somewhat subjective. Could the authors clarify the basis or thresholds used to define “excellent” (>80%) and “good” (>70%) coverage?
6. Meta-proteomics is more frequently written as “metaproteomics”. In addition, metaproteomics is usually used to analyze complex communities, so I would recommend that the authors use proteomics throught the text.
7. Line 85, The phrase “and were somewhat uniformly distributed” is not a scientifically rigorous description. Based on the data presented, there does not appear to be a clear or consistent distribution pattern.

Reviewer #2 (Remarks to the Author):

Summary --

The authors present an interesting and impactful set of in vitro co-culture experiments, where they measure meta-proteomic and metabolomic responses to pairwise co-cultures, relative to monocultures. They show data from 104 pairwise co-cultures of 15 gut bacterial isolates, with replicates for each co-culture and monoculture condition. They find frequent proteome reorganization to co-culture partners and cross-feeding interactions. The novelty of this study is in its combination of meta-proteomics and metabolomics across a wide variety of microbe-microbe interactions. The conclusions are well supported by the data. Many of the findings are in line with prior in vitro work with similar sets of isolates, but other findings are more novel, like the observation that changes in metabolite abundances are not strongly coupled to changes in the proteome. These results and data will be useful resources to the microbial ecology community. I commend the authors on a rigorous set of experiments, elegant analyses, and a clearly-written manuscript. I outline a few minor points below.

Major comment --

The authors should include a section in the discussion that highlights the novelty of this work relative to other in vitro microbe-microbe interaction studies (this is becoming a crowded field, with lots of papers published every month). Along this line, I see that the authors mention building CarveMe models of isolates, but I didn't see any mention of metabolic modeling in the results. Did you try to apply FBA to these co-culture systems to further validate/explore mechanisms? Even if you only used this approach to look at a few co-culture pairs, this could be an interesting addition to the current set of results, which could further increase the impact/novelty of your work.

Minor points --

line 13: You mention 'trophic levels', but would it be better to use the term 'guilds'? When I think of trophic levels in macro-systems, I think of primary producers (plants), primary consumers (herbivores), and secondary consumers (predators).

Trophic relationships often involve predation/consumption, whereas these kinds of interactions are rare between bacteria. I see bacteria-bacteria interactions as being predominantly driven by competition for metabolites, which seems to be within the same 'trophic' layer (you'd need to include phage, protists, invertebrates, etc., to get higher trophic levels).

line 26: change 'mouse' to 'mice'

lines 199-210: This is a great section. I like how you were able to explain the discrepancy between your results and prior work, highlighting the context-dependency of the nutrient environment.

lines 271-280: I think this is one of the more interesting/novel aspects of your study -- that you do not find a strong association between changes in metabolite levels and proteome composition. This does suggest a strong metabolite-control over metabolic activity in these systems (i.e., the availability of nutrients governs the fluxes through reactions more than the abundances of enzymes). This bodes well for methods like FBA (see major comment above).

Figure 6: This is another relatively novel aspect of your study to highlight -- the clustering of genes to identify putative functions to non-annotated proteins. Maybe you could explore this a bit more in the results/discussion?

********************END********************

Version 1:

Decision Letter:

21st January 2026

Dear Dr. Patil,

Thank you for submitting your revised manuscript "Interspecies interactions drive bacterial proteome reorganisation and emergent metabolism" (NATECOLEVOL-25051557A). It has now been seen again by the original reviewers and their comments are below. The reviewers find that the paper has improved in revision, and therefore we'll be happy in principle to publish it in Nature Ecology & Evolution, pending minor revisions to satisfy the reviewers' final requests and to comply with our editorial and formatting guidelines.

If the current version of your manuscript is in a PDF format, please email us a copy of the file in an editable format (Microsoft Word or LaTex)-- we can not proceed with PDFs at this stage.

If you have not done so already, please ensure that you also email us a completed copy of the Reporting summary :

Reporting summary: https://www.nature.com/documents/nr-reporting-summary.pdf

We are now performing detailed checks on your paper and will send you a checklist detailing our editorial and formatting requirements in about a week. Please do not upload the final materials and make any revisions until you receive this additional information from us.

Thank you again for your interest in Nature Ecology & Evolution. Please do not hesitate to contact me if you have any questions.

[redacted]

Reviewer #1 (Remarks to the Author):

I appreciate the substantial effort the authors have made to address the revision comments. The responses and discussion have been enjoyable to follow, and I believe the manuscript will be of interest to readers. Overall, I consider the manuscript suitable for acceptance, with only one minor issue to be addressed: in the revised Extended Data Figure 2G, some colors are repeated (for example, Bt vs Pc, Bu vs Pm, and Bv vs Rg), which may cause confusion and should be adjusted.

Reviewer #2 (Remarks to the Author):

The authors have done a great job on the revision. I have no further comments or concerns.

Open Access This Peer Review File is licensed under a Creative Commons Attribution 4.0 International License, which permits use, sharing, adaptation, distribution and reproduction in any medium or format, as long as you give appropriate credit to the original author(s) and the source, provide a link to the Creative Commons license, and indicate if changes were made.
In cases where reviewers are anonymous, credit should be given to 'Anonymous Referee' and the source.
The images or other third party material in this Peer Review File are included in the article's Creative Commons license, unless indicated otherwise in a credit line to the material. If material is not included in the article's Creative Commons license and your intended use is not permitted by statutory regulation or exceeds the permitted use, you will need to obtain permission directly from the copyright holder.
To view a copy of this license, visit https://creativecommons.org/licenses/by/4.0/

Reviewer #1

The study examined 104 pairwise co-cultures of 15 gut bacteria, revealing diverse ecological interactions—mainly competition, amensalism, and exploitation. It offers important advantages, particularly from a proteomics-informed systems ecology viewpoint, highlighting the molecular scale of ecological interactions that cannot be captured by genomic data alone.
The data and results reported are extensive, and the analysis progresses from broad profiling to deeper mechanistic insights, offering multiple perspectives and novel observations. Overall, it presents a valuable contribution to the field. I do, however, have several comments and suggestions that I believe could further improve the clarity, robustness, and impact of the manuscript.

We thank the Reviewer for their time and valuable feedback. We have revised the manuscript to address the points raised, as detailed below.

Major comments

1. Out of 768 samples, 106 were excluded (~14%) is notable and warrants clarification. This is a substantial proportion—can the authors clarify the reasons for such sample loss? Could this be due to competitive suppression of growth, technical issues in proteomics processing, or contamination during culturing?

Thank you for raising this. It's due to a combination of factors. Starting from 8 complete 96-well plates (n=768), the number of excluded samples is a summation of the following factors:

- One row of (n=12) samples contained low amounts of peptides after digestion. We suspect that this was due to inadvertent omission of trypsin addition to these samples. These were not measured. Another 3 samples showed low signal and no identifications, possibly due to an injection or another technical issue.
- We applied stringent filtering based on the number of precursors IDs and the fraction of dataset-wide proteotypic precursor IDs attributable to the species expected in the samples. This removed an additional 91 samples.
- One of our cryostocks (*Bifidobacterium longum*) was later found to be contaminated during routine QC check. All remaining samples containing this species (n=46) were excluded, reducing the set of species from 16 to 15.
- 8 co-cultures displayed unusually high ODs. While this is most likely due to a measurement error such as an air bubble, this could stem from contamination, so we decided to be conservative and exclude those samples.

These steps are explained in the Methods section. While we agree that this is a considerable part of the data, it's not atypical for high-throughput experiments, and we deliberately used stringent quality filtering, as the number of replicates included in the study design (n=17 for mono-cultures and n=4 for co-cultures) allowed for selecting only the highest-quality data. Most of the removed/missing samples (78 of 106) contained the species *Bifidobacterium longum* and the impact of sample filtering on the remaining dataset is therefore small.

2. Figure 1H is very interesting, as it illustrates how certain species tend to dominate in co-cultures, reflecting their growth advantages—at least under the specific culture medium used in this study. However, this information could be presented more clearly and intuitively. I suggest adding a 14 × 14 matrix of stacked bar plots or pie charts (to replace Figure S1F), where each cell visualizes the relative protein biomass contribution of each species within a given co-culture, similar in style to Figure S1D. This would provide a more immediate and accessible visual impression of species dominance patterns across co-culture conditions.

Thank you for this suggestion. We have created the requested plot (New Extended Data Figure 2E). We have also improved the bar plot (Extended Data Figure 2G) which now includes error bars that illustrate the level of noise (standard deviation) of the relative abundance measurements.

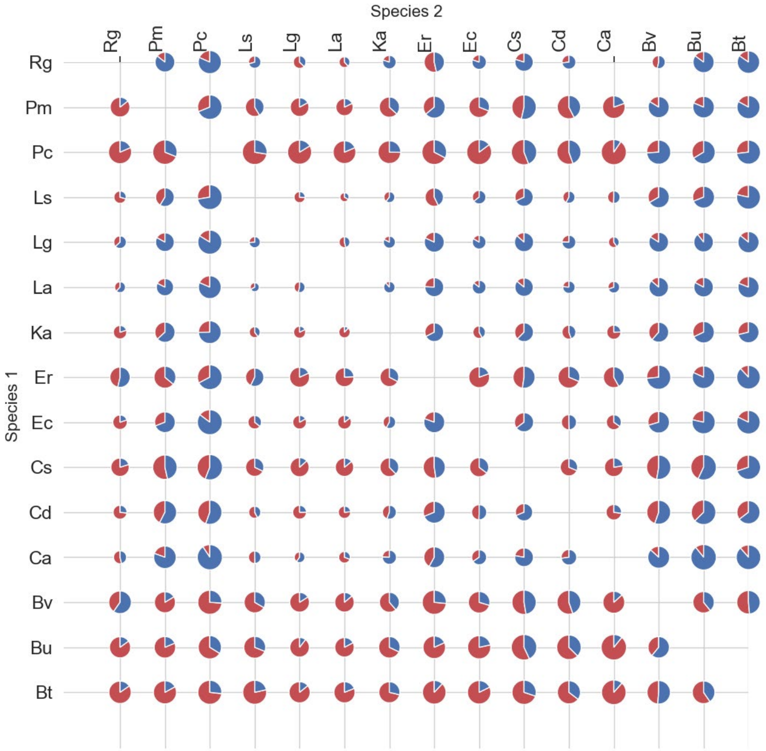

EDFig 2e: Relative abundance of species across co-culture conditions. The size of the pie chart reflects the total co-culture OD.

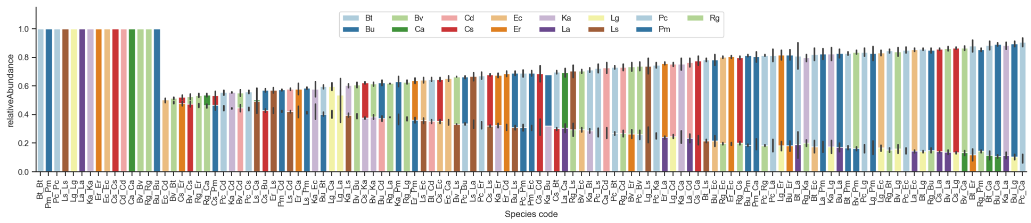

EDFig 2g: Relative abundances for all 104 co-culture pairs, errorbars indicate the standard deviation.

3. Figure 1K is also interesting, I've recently came across a study that discussed co-culture interactions by colony shapes that two species form: https://microbiomejournal.biomedcentral.com/articles/10.1186/s40168-025-02156-0.
The authors have concluded a similar stacked column bar chart. It may be worthwhile for the authors to consider comparing and discussing their findings in the context of this study, particularly regarding how different experimental platforms (e.g., colony-based vs. proteomics-based) may reveal similar or complementary interaction patterns.

This paper (Zhu et al 2025) was published during the review process; we read it with great interest and had already planned to discuss it. A comparison of the frequencies of the different interaction types across is shown below and in New Extended Data Figure 2H. Our results agree closely with Venturelli 2018, displaying competition, exploitation and amensalism as roughly evenly distributed, major interaction types. Zhu et al. 2025 detect fewer instances of exploitation and higher instances of neutralism and commensalism.

Zhu 2025 use the same media (mGAM) as we do, but in solid (agar form). Venturelli 2018 use liquid Anaerobic Basal Broth (ABB), a largely similar medium with fewer animal-derived components.
Overall, these three studies are highly orthogonal in their methodology, using proteomics, 16S sequencing and colony image analysis as readouts, but produce largely similar ecological interaction types. The slight disagreement between Zhu 2025 and our study/Venturelli 2018 could be due to the use of solid media, as it is known that inter-species interactions can dramatically differ between solid and liquid media (e.g. see Blasche et al. Nature Microbiology, 2018).

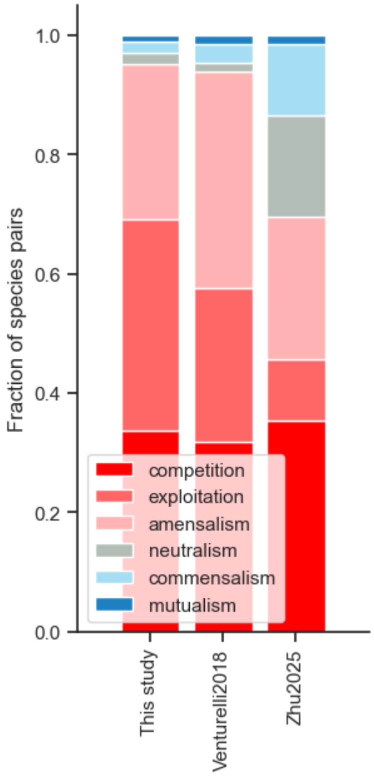

ED Fig 2h: Comparison of frequencies of ecological interaction types with other studies. Venturelli et al 2018 used liquid cultures in Anaerobic Basal Broth, while Zhu et al 2025 used solid mGAM medium.

On an additional note, something I was wondering when reading the above paper: proteomics could be adapted for spatially resolved analysis in plate-based co-culture systems. Given the sensitivity of modern proteomic workflows to low biomass, would it be feasible to perform protein extraction specifically at the colony–colony interface to capture spatial interaction zones? Such spatially resolved metaproteomics could potentially offer unique insights into the localized molecular responses that occur during direct microbial contact.

Yes, absolutely. Going forward, we have plans to explore the spatial organisation of gene expression in gut microbial communities. However, for this study, we consider these types of experiments out of scope.

4. Before mentioning results related Figure 1J-K, the authors should first clearly define how negative an positive interactions, as well as the five different types of ecological interactions – how were these quantified from the dataset? Clarifying this will enhance the reader's ability to interpret Figure 1J–K, assess the biological relevance of the findings, and understand the ecological implications of the observed patterns.

Thank you for raising this point. The quantification of relative abundances from proteomics data relies on several filtering and normalisation steps. These are explained in the Methods section (reproduced below) and we now additionally include the analysis code as a Jupyter notebook in

the Mendeley Data repository (https://data.mendeley.com/preview/6djkbgs22f?a=69e98f31-39f4-4b35-bf7f-b2f0af3e80fe).

> We used the peptide report from stage 1 (above), filtered for proteotypic peptides (across all species) and q-value and Quantity.Quality filters as before. For each species, highly complete precursors were selected (identified in >90% of samples) and missing values imputed using median imputation. The sum of non-normalised precursor quantities (column Precursor.Quantity) was used as proxy of relative species abundance which was divided by the same value obtained for the mono-culture of that species to correct for the fact that an identical amount of injected peptides will produce different intensity sums for each species (e.g. due to the quality of the annotated proteome or physicochemical peptide properties). For each co-culture sample, the quantities obtained for each species were divided by the sum of both to obtain relative abundances for each sample. These were then scaled by the total OD600 of the co-culture to obtain absolute abundances of each species in the co-culture in OD units.

The applied fold-change threshold (10%) is stated in Results section and figure legend, along with a definition of each ecological interaction type (e.g. 'amensal (one species inhibited, the other unchanged)').

It is also important to clarify whether samples excluded due to poor proteomic quality may have had low protein yield because of underlying ecological factors, such as limited growth or biomass production in specific co-culture conditions. If so, this could introduce bias into the conclusions presented in Figure 1K, as certain interaction types, especially those associated with low-abundance, may be systematically underrepresented.

This is a valid point, thank you. Most of the removed/missing samples (78 of 106) contained the species *Bifidobacterium longum* which was completely removed from the dataset due to contamination of the preculture. Other than that, except for the Rg-Pv pair (n=0 replicates passing filters), we had enough (typically n=17 replicates for monocultures and n=4 replicates for co-cultures; minimum number of replicates per co-culture n=2) replicates in the dataset so that exclusion of the other filtered samples did not result in substantial data loss at the species level.

We would also like to highlight that low abundance of one partner species in co-culture would not have led to that sample being filtered out since the number of peptide identifications required was permissive enough for monocultures and absence of a species does not affect the sample purity score.

We only used high-abundant, high-quality peptides to compute abundances and we imputed missing values for this analysis, minimising the occurrence and impact of missing data at the peptide level.

Overall, sample exclusion is not tightly linked to the species pair (and thereby the ecological interaction type), resulting in minimal bias from this source.

Were any statistical criteria or thresholds applied in combination with the ecological interaction typing? For example, were detections on changes of biomass supported by significance testing? Clarifying this would strengthen confidence in the robustness and reproducibility of the interaction classifications.

We have performed statistical significance testing for the ecological interactions, by testing the null hypothesis that mean absolute abundances in co- and monoculture are the same (via two-sided Student's t-test). After correcting for multiple testing (Benjamini-Hochberg method), 172 out of 179 interactions which previously passed our 10% fold-change threshold were statistically significant ($p_{adj}<0.05$). We now report the p-values in the Supplementary Data and have included the code in the Mendeley Data repository. Further, maximum likelihood (estimated as the mean) is appropriate basis for classification of ecological interactions, as significance testing only tests the probability of the null hypothesis being false but not the probability of it being correct. While the choice of thresholds is always somewhat subjective, we believe that our choice (10% change) is meaningful both in biological terms, as well as in the context of the noise level of the data (typical standard deviation=5%).

5. The manuscript states that in 1,163 instances (0.25% of the dataset), a protein was only identified in co-culture but not in mono-culture, and these were classified as hits. Could the authors clarify how "only identified in co-culture" was defined? Specifically, was identification required in all technical replicates of the co-culture and absent in all replicates of the mono-culture, or was detection in any single replicate considered sufficient? It is known that in proteomics, even technical replicates of the same sample may yield partially inconsistent identifications. Proper statistical handling and reproducibility filtering are necessary to rule out stochastic detection effects.

This is a very valid point and to address this stringently we conducted an additional control experiment. Three co-cultures containing gram-positive and -negative species at different ratios spanning the previously observed range (1:1 to 1:9) were measured in quadruplicates, as well as the same number of corresponding mono-cultures. To isolate LC-MS matrix effects in the absence of biological proteome changes, we mixed mono-culture samples in the same ratio (co-measurement controls). Additionally, we mixed mono-cultures in the same ratio before protein extraction (co-extraction controls) to detect potential artefacts introduced during sample preparation. The results of this experiment are shown in the new Extended Data Fig. 1, reproduced below.

Using default settings, some artefact hits (i.e. attributable to co-extraction or co-measurement) were detected in the controls (panel B). The benchmark dataset was used to optimise the data processing and we have thus implemented the following additional, stricter, filters during data processing: at the precursor level we now require Quantity.Quality>0.85 (previously >0.75) and a Precursor.Quantity>10000. We also removed precursors not identified in mono-culture as these carry no value for protein abundance quantification but can in rare cases lead to unexpected results when used with maxLFQ. At the protein level, we now require at least two replicates with non-NA values (i.e. ≥50% data completeness) and at least two precursors per protein (panel C). This substantially reduces the number of artefacts (panel B).

Interestingly, co-extraction results in more artefact hits than co-measurement, indicating that LC-MS matrix effects are small compared to variation introduced during the sample preparation, which could be due to secreted factors. However, the hits in co-extraction samples show only small overlaps with the co-culture hits, meaning that any variation in co-culture is not explained by co-extraction artefacts (Panel D+E).

Based on these stricter filtering criteria, we have re-analysed the main dataset. Heatmaps and other analyses that illustrate fold-changes rather than binary hit classifications, are not strongly affected by this change. The overall number of hits is now lower (28,113 hits versus 60,066), however, the key conclusions of the paper are not affected, In the opposite, in several instances, the stricter filtering resulted in stronger biological signals. E.g., we now identify 31 enriched terms in the collective set of hits (up from 7) and 49% of protein clusters are enriched in at least one term (up from 18%). Full statistics for each protein (p-values, fold-changes, number of datapoints, number of underlying peptides) are reported in Supplementary Table 2+3.

Regarding protein hits only detected in co-culture, these proteins got filtered out in the new analysis as it is not possible to tell from the new Benchmark dataset how error prone this type of hit is. As this affected only a few hundred instances (0.25% of the dataset), this does not alter any conclusions or the overall structure of the data.

Overall, these revisions strengthen our manuscript and provide robust insights into proteomic responses in co-cultures.

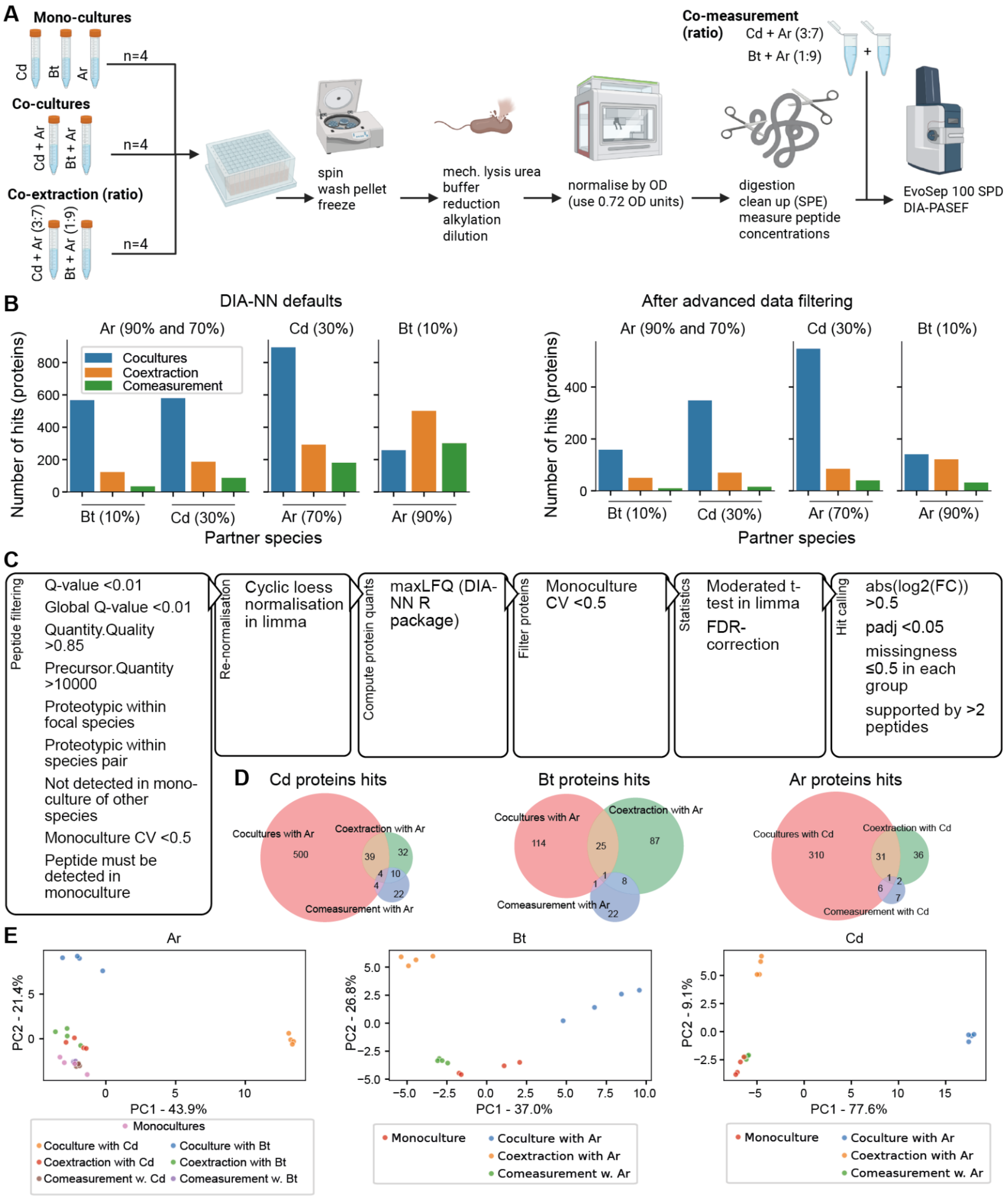

EDFig 1: Benchmark dataset controls for matrix effects

(A) Overview of benchmark experiment workflow. Three co-cultures containing gram-positive and -negative species at different ratios spanning the previously observed range (1:1 to 1:9) were measured in quadruplicates, as well as the same number of corresponding mono-cultures. To isolate LC-MS matrix effects in the absence of biological proteome changes, we mixed mono-culture samples in the same ratio (co-measurement controls). Additionally, we mixed mono-cultures in the same ratio before protein extraction (co-extraction controls) to detect potential artefacts introduced during sample preparation. Created in BioRender. Kamrad, S. (2026) https://BioRender.com/13dsg6p

(B) Number of hits with DIA-NN default parameters (i.e., using protein quantities provided by DIA-NN without additional filtering, right) versus after filtering (see panel C, right). Hits are defined relative to mono-culture controls, abs(log_2(co-/mono-culture)>0.5 and p_{adj}<0.05.

(C) Implemented data filtering steps which successfully remove most coextraction and comeasurement artefacts.
(D) Overlap of hit proteins across co-culture and control conditions.
(E) Principal component analysis of proteome profiles, each datapoint represents an individual sample.

6. Supplementary Figure 6, it's interesting to see GABA responses, wondering if the authors checked the protein levels related to GABA pathways in the co-cultures. Proteins involved in GABA pathways can be annotated using eggNOG, KEGG etc.

Gut-bacterial GABA as an effector in the gut-brain axis has been an active topic of research. *Bacteroides* are known to be the major producers of GABA under physiological conditions and the four-gene operon to do so is well described (Otaru 2021 *Frontiers in Microbiology*). This is primarily thought to be a mechanism to stabilise/increase intracellular pH, similar to textbook amino acid decarboxylation reactions originally described in *E. coli.* Accordingly, in co-cultures involving B*. uniformis* (highlighted in the original SFig6) we also observe a pH-dependence, with mild acid stress inducing GABA production. Interestingly, *B. theta* showed overall higher GABA levels but no correlation of GABA production with pH.

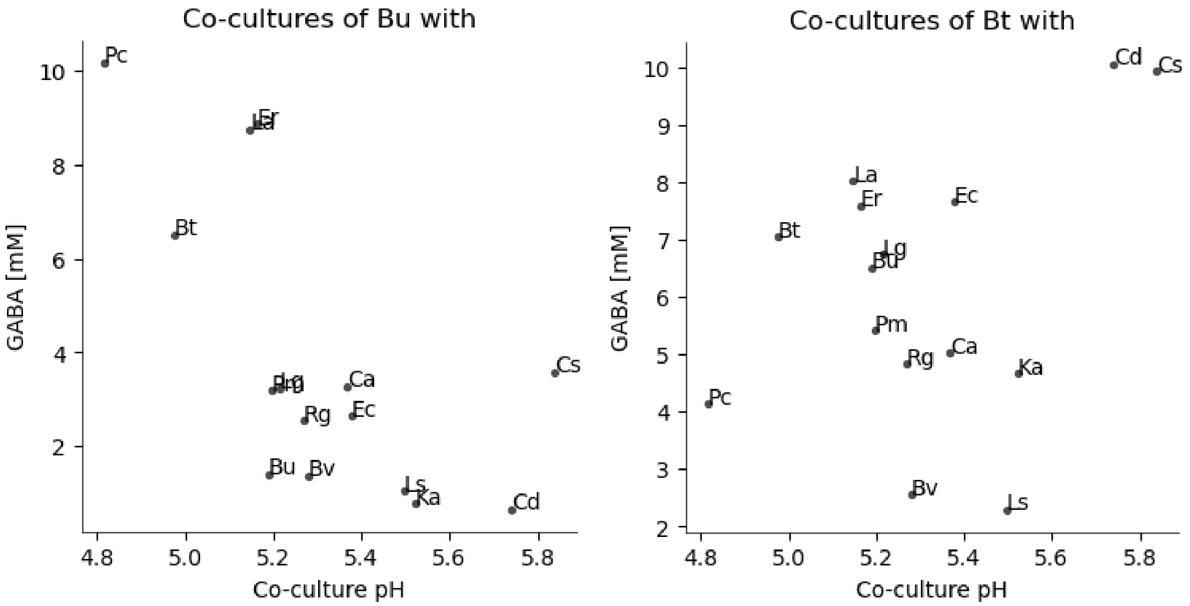

ED Fig 7d: GABA concentrations in co-cultures involving *B. thetaiotaomicron* (left) and *B. uniformis* (right), depending on co-culture pH. *B. uniformis* cultures show increases GABA concentrations with decreasing pH, while no such relationship is observed for *B. thetaiotaomicron* co-cultures over a similar pH range.

The GAD system is well characterised in *B. thetaomicron* and consists of the following four genes:
Glutamate dehydroxylase (gadB, BT_2570, Q8A4M9),
Glutaminase (glsA, BT_2571, Q8A4M8)

Potassium channel (BT_2572, Q8A4M7)
Glutamate/GABA antiporter (BT_2573, Q8A4M6)

Orthologues were mapped in *B. uniformis* (OrthoFinder). Only gadB and glsA were captured in the proteomics dataset and indeed showed concordant expression that indicates upregulation under acid stress. *B. thetaiotaomicron* also showed strong regulation of these proteins in concordance with GABA production. Interestingly, the two *Clostridia C. difficile and C. sporogenes* showed the highest upregulation of the enzymes and also the highest GABA levels, despite no rise in pH.

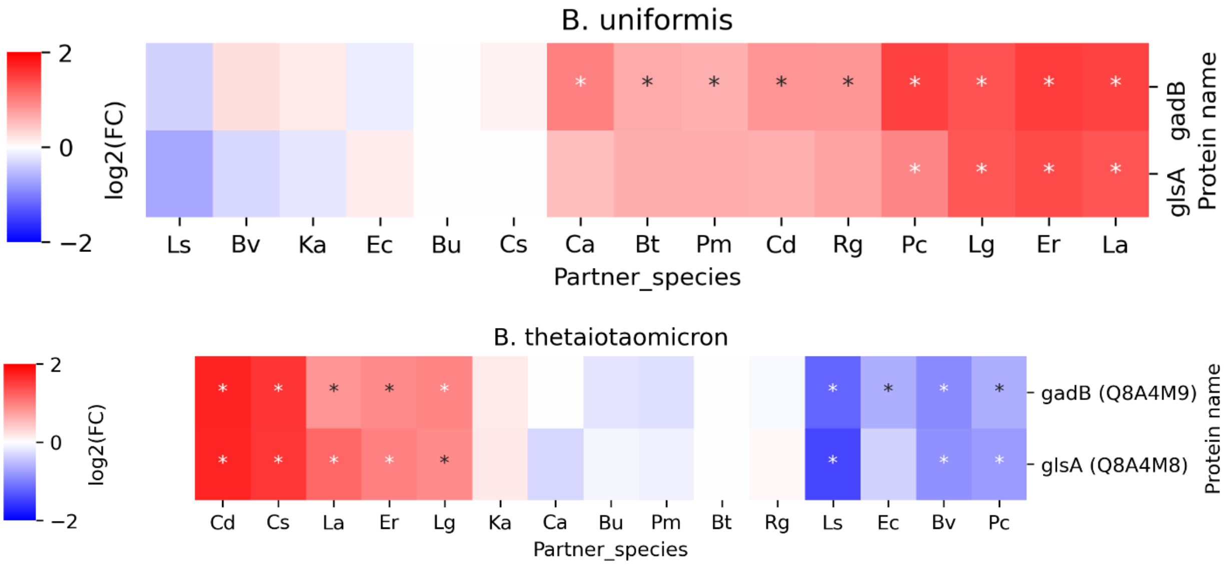

ED Fig 7e: Regulation of GAD operon genes recapitulates observed changes in GABA concentrations, including an upregulation in *B. thetaiotaomicron* in co-culture with *C. difficile* and and *C. sporogenes*. Protein identifiers in *B. thetaiotaomicron*: glsA - Q8A4M8; gadB - Q8A4M9. Protein identifiers in *B. uniformis*: glsA - UPI0006C4376F; gadB - UPI00015BEEBA.

In summary, GABA production by *Bacteroides* appears to be under significant enzyme control (in contrast to the production of polyamines by *E. coli*, as shown in Fig 4F). *B. theta* and *B. uniformis* experience similar pH ranges in co-cultures however only *B. theta* responds by upregulating GABA production. What triggers GABA production in *B. theta* in response to the *Clostridia* is an open question. We have included the above plots in Extended Data Fig 7 D+E and would like to thank the reviewer for suggesting these analyses.

7. The response of ‘genetic dark matter’ related proteins (Fig6D) is intriguing and highlights a substantial proportion of co-culture-responsive proteins that remain functionally unannotated. This section would benefit from a more hypothesis-generating exploration of this ‘genetic dark matter’ rather than ending with general statements. For example, it is known that eggNOG annotates proteins not only using KEGG but also other methods such as COG, Pfam , and GO. Are any of these poorly annotated proteins characterized through non-KEGG methods? In

addition, some recent studies have attempted to functionally profile microbiome 'dark matter', could any of these approaches be used to further interpret the co-culture responsive yet uncharacterized proteins in this study?

Thank you for raising this valid point. We had used the eggNOG mapper as well as the KEGG annotation tool to retrieve diverse functional annotations and these are all contained in Supplementary Data 3 ('Protein metadata'). Of the 12,016 proteins in the dataset for which either no KEGG KO was available or it was 'uncharacterized', only 187 (1.56%) had GO annotations (via eggNOG). 7,839 were assigned COG category other than S ('unknown function'), but given the coarse-grained nature of COG annotation, this is of limited value. Pfams are similarly of limited use as this is primarily an unsupervised clustering approach that contains many domains of unknown function and broad functional categories. This is not to say that these tools are not useful for generating hypotheses about protein function, but we believe that KEGG KOs and pathways most accurately reflect the annotation status of bacterial proteins.

8. The authors should provide reviewer access to the proteomics raw dataset. I wasn't able to find it among the submitted files.

The proteomics data is publicly available via PRIDE as indicated in the Data Availability section:

> "The mass spectrometry proteomics data have been deposited to the ProteomeXchange Consortium (http://proteomecentral.proteomexchange.org) via the PRIDE partner repository [116] with the dataset identifier PXD055395."

We now provide additional code to process the data and intermediate files (Mendeley Data), as well as Source Data for the figures.

Minor comments

1. Line 45, These 45 include abundant and prevalent commensal species from four phyla, better expand what these four phyla are.

Thank you, the names of the phyla have been specified: "(Firmicutes, Bacteroidetes, Proteobacteria and Actinobacteria)"

2. From a logical perspective, in the first result paragraph of lines 43–52, readers may wonder about the deeper rationale behind the species selection—for example, whether the selection was linked to diverse ecological interactions. Was the choice of species informed by a pre-designed consideration of potential interactions?

Thank you for highlighting this, we have added a sentence to explain the rationale: "This diverse set includes species with complementary nutrient requirements and known cross-feeding interactions [18], as well as understudied interactions like those between probiotics and resident microbiota. "

3. Line 57, The term “concatenated library-free approach” may be difficult to understand for readers who are not familiar with proteomics. I suggest the authors consider rephrasing it in a more accessible way or briefly explaining the concept upon first mention, to improve clarity and ensure broader comprehension.

Thank you, we have re-phrased this as ‘a library-free search with a combined fasta-database of all 15 species’.

4. Line 68, the statement “covers the vast majority of the proteome by mass” lacks direct quantitative evidence from this study. To avoid overstatement, tone down as “is expected to capture the majority of the proteome by mass”.

Thank you, we were referring here to a general characteristic of proteomics datasets and have re-phrased accordingly.

> Our dataset, like other proteomic datasets, is expected to capture most of the proteome by mass, since highly abundant proteins are much more likely to be measured. E.g., Schmidt et al [50] estimated that the 55% of *Escherichia coli* ORFs detected in their dataset cover 95% of the proteome by mass.

5. Lines 72-75. The terms “excellent coverage” and “good coverage” for different functional categories (e.g., enzymes, ribosomal proteins, transcription factors) are somewhat subjective. Could the authors clarify the basis or thresholds used to define “excellent” (>80%) and “good” (>70%) coverage?

We have removed these subjective terms as suggested.

6. Meta-proteomics is more frequently written as “metaproteomics”. In addition, metaproteomics is usually used to analyze complex communities, so I would recommend that the authors use proteomics throughout the text.

While we do not necessarily agree that ‘metaproteomics’ should be reserved only for natural communities, we have adapted the text to primarily use ‘proteomics’ as suggested.

7. Line 85, The phrase “and were somewhat uniformly distributed” is not a scientifically rigorous description. Based on the data presented, there does not appear to be a clear or consistent distribution pattern.

Thank you, we agree and have re-phrased to ‘no clear distribution pattern’.

Reviewer #2

The authors present an interesting and impactful set of in vitro co-culture experiments, where they measure meta-proteomic and metabolomic responses to pairwise co-cultures, relative to monocultures. They show data from 104 pairwise co-cultures of 15 gut bacterial isolates, with replicates for each co-culture and monoculture condition. They find frequent proteome reorganization to co-culture partners and cross-feeding interactions. The novelty of this study is in its combination of meta-proteomics and metabolomics across a wide variety of microbe-microbe interactions. The conclusions are well supported by the data. Many of the findings are in line with prior in vitro work with similar sets of isolates, but other findings are more novel, like the observation that changes in metabolite abundances are not strongly coupled to changes in the proteome. These results and data will be useful resources to the microbial ecology community. I commend the authors on a rigorous set of experiments, elegant analyses, and a clearly-written manuscript. I outline a few minor points below.

We would like to thank the reviewer for their efforts and the positive and constructive feedback. We have revised the manuscript in response to the points raised, as outlined below.

Major comment --

The authors should include a section in the discussion that highlights the novelty of this work relative to other in vitro microbe-microbe interaction studies (this is becoming a crowded field, with lots of papers published every month). Along this line, I see that the authors mention building CarveMe models of isolates, but I didn't see any mention of metabolic modeling in the results. Did you try to apply FBA to these co-culture systems to further validate/explore mechanisms? Even if you only used this approach to look at a few co-culture pairs, this could be an interesting addition to the current set of results, which could further increase the impact/novelty of your work.

Thank you for these suggestions. We have added Extended Data Figure 2H which compares our results to other in vitro interaction studies by Zhu et al (July 2025) *Microbiome* and Venturelli (2018) *MSB*. This analysis is discussed in the Results:

> These results are in good agreement with other, methodologically complementary studies (different readouts and media) of pairwise interactions among gut bacteria [11,16] (**Extended Data Fig 2H**).

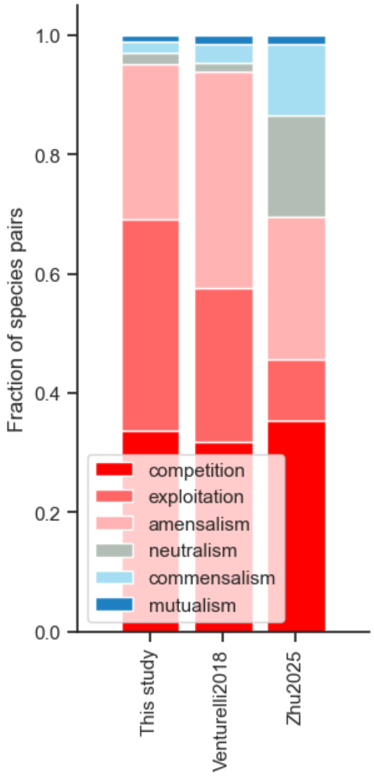

ED Fig 2h: Comparison of frequencies of ecological interaction types with other studies. Venturelli et al 2018 used liquid cultures in Anaerobic Basal Broth, while Zhu et al 2025 used solid mGAM medium.

The novelty of our study lies in deep molecular analysis of co-cultures that goes beyond the typical growth readouts and provides new insights into the molecular response of species to the presence of others. While we agree that metabolic modelling could be useful, is beyond the scope of the present study focussed on proteomics and metabolomics of defined cocultures at unprecedented scale.

Minor points --

line 13: You mention 'trophic levels', but would it be better to use the term 'guilds'? When I think of trophic levels in macro-systems, I think of primary producers (plants), primary consumers (herbivores), and secondary consumers (predators). Trophic relationships often involve predation/consumption, whereas these kinds of interactions are rare between bacteria. I see bacteria-bacteria interactions as being predominantly driven by competition for metabolites, which seems to be within the same 'trophic' layer (you'd need to include phage, protists, invertebrates, etc., to get higher trophic levels).

Thank you for highlighting these distinctions, we have removed ‘across several trophic levels’ from the sentence. The revised section reads as below:

> We here use gut bacterial isolates to probe the molecular basis of interspecies interactions. Ecological interactions among gut bacteria comprise complex competitive and cooperative interactions [19–23].

line 26: change 'mouse' to 'mice'

Thank you, done.

lines 199-210: This is a great section. I like how you were able to explain the discrepancy between your results and prior work, highlighting the context-dependency of the nutrient environment.

Thank you.

lines 271-280: I think this is one of the more interesting/novel aspects of your study -- that you do not find a strong association between changes in metabolite levels and proteome composition. This does suggest a strong metabolite-control over metabolic activity in these systems (i.e., the availability of nutrients governs the fluxes through reactions more than the abundances of enzymes). This bodes well for methods like FBA (see major comment above).

Thank you, we have expanded the Discussion to better highlight this observation. We also highlight other instances where enzyme control appears important (in the case of GABA, see response to Reviewer 1 point 6).

> The observed metabolic interactions are closely intertwined with the proteome, evident in the strong enrichment of enzymes and transporters among the responsive proteins. Some metabolic changes correlated directly with enzyme abundances, e.g., GABA synthesis in *Bacteroides* and arginine biosynthesis in response to the arginine consumer *C. aerofaciens*. In other cases, metabolic changes appear to occur without changes in enzyme abundance, e.g., polyamine synthesis. This is congruent with pathways being regulated by either enzyme availability (enzyme control) or by metabolite availability (metabolic control). Thus, proteome and metabolome serve as complementary readouts, with enzyme regulation data indicative of metabolic changes directed by the cellular regulatory machinery.

Figure 6: This is another relatively novel aspect of your study to highlight -- the clustering of genes to identify putative functions to non-annotated proteins. Maybe you could explore this a bit more in the results/discussion?

Thank you for highlighting this. We consider these points in the Discussion:

> If large proteomic datasets are available, co-variation analysis can be a powerful way of gene functional annotation, which at least in human [95] and yeast [96], outperforms other strategies of functional annotation of unknown protein function, and is a particularly attractive strategy for annotating gene function in species that are difficult to manipulate genetically. We have here shown that systematic co-culture proteomics cluster into functionally enriched sets of proteins. At the same time, our case study of *S. copri* shows that many strongly and specifically regulated protein clusters are entirely un-annotated to date, thereby identifying priority unstudied genes in the community context. Our co-culture proteomics data is thus a useful complementation to other current systematic functional genomics efforts in the microbiome space, e.g. involving forward genetic screens through transposon mutagenesis [97,98].

Reviewer #1:

Remarks to the Author:

I appreciate the substantial effort the authors have made to address the revision comments. The responses and discussion have been enjoyable to follow, and I believe the manuscript will be of interest to readers. Overall, I consider the manuscript suitable for acceptance, with only one minor issue to be addressed: in the revised Extended Data Figure 2G, some colors are repeated (for example, Bt vs Pc, Bu vs Pm, and Bv vs Rg), which may cause confusion and should be adjusted.

We would like to thank the reviewer for their efforts, the fast turnaround time and the constructive feedback.

Thank you for spotting the issue with the colour scheme. We have rectified this, as shown below.

g

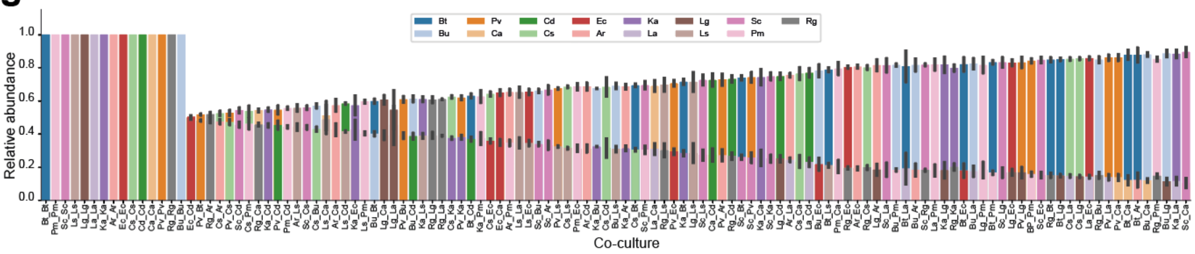

Reviewer #2:

Remarks to the Author:

The authors have done a great job on the revision. I have no further comments or concerns.

Thank you for your encouraging comments and constructive feedback.
